# Occupational differences in COVID-19 hospital admission and mortality risks between women and men in Scotland: a population-based study using linked administrative data

**DOI:** 10.1136/oemed-2024-109562

**Published:** 2025-04-29

**Authors:** Serena Pattaro, Nick Bailey, Chris Dibben

**Affiliations:** 1 Scottish Centre for Administrative Data Research (SCADR), School of Social and Political Sciences, University of Glasgow, Glasgow, UK; 2 Scottish Centre for Administrative Data Research (SCADR), School of Geosciences, University of Edinburgh, Edinburgh, UK

**Keywords:** COVID-19, Mortality, Occupational Health, Occupational Groups, Public health

## Abstract

**Objectives:**

Occupations vary with respect to workplace factors that influence exposure to COVID-19, such as ventilation, social contacts and protective equipment. Variations between women and men may arise because they have different occupational roles or behavioural responses. We estimated occupational differences in COVID-19 hospital admission and mortality risks by sex.

**Methods:**

We combined (1) individual-level data from 2011 Census with (2) health records and (3) household-level information from residential identifiers, using a Scottish cohort of 1.7 million adults aged 40–64 years between 1 March 2020 and 31 January 2021. We estimated age-standardised COVID-19 hospital admission and mortality rates, stratified by sex and occupation. Cox proportional hazards models were adjusted for pre-pandemic health and occupational exposure factors, including interaction effects between occupation and sex.

**Results:**

Women had lower age-standardised COVID-19 hospital admission and mortality rates than men. Among women, adjusted death risks were lowest for health professionals, and those in associate professional and technical occupations (paramedics and medical technicians), with the latter supported by results from the interaction model. Among men, elevated adjusted admission and death risks were observed for large vehicle and taxi drivers. Additionally, admission risks remained high among men in caring personal services (including home and care workers), while elevated risks were observed among women in customer service occupations (call centre operators) and process, plant and machine operative roles (assemblers and sorters).

**Conclusions:**

Occupational differences in COVID-19 hospital admission and mortality risks between women and men highlight the need to account for sex differences when developing interventions to reduce infections among vulnerable occupational groups.

**WHAT IS ALREADY KNOWN ON THIS TOPIC**

- There are large differences in the risk of severe COVID-19 outcomes (hospitalisation and mortality) between women and men.
- While most population-based studies have reported occupational differences in COVID-19 mortality risks, a small proportion of these studies have investigated hospital admission risks.
- Additionally, few studies have controlled for prepandemic health or explored occupational exposure factors when assessing these risks.

**WHAT THIS STUDY ADDS**

- We estimated COVID-19 hospital admission and mortality risks for different occupational groups by sex in Scotland during the period between March 2020 and January 2021.
- We found lower age-standardised COVID-19 hospital admission and mortality rates for women, with disparities across different occupational groups, such as higher mortality rates for women in process and machine operative roles and men in transportation and elementary services.
- Adjusting for socioeconomic, pre-pandemic health and occupational exposure factors reduced the association between COVID-19 admission and mortality risks across most occupational groups. While a similar occupational gradient emerged for admission risks between women and men, there were differences for mortality, with women in less disadvantaged occupations (eg, health professionals) showing lower risks and men in more disadvantaged occupations (eg, large vehicle and taxi drivers) exhibiting higher risks.

**HOW THIS STUDY MIGHT AFFECT RESEARCH, PRACTICE OR POLICY**

- Reducing sex discrepancies in future respiratory pandemics requires coordinated interventions addressing gaps in the provision of protective equipment and training among health professionals and care workers, and targeting high-risk occupational groups working in transportation and personal services sectors.

## Introduction

 The COVID-19 pandemic had unequal effects on working-age women and men, with higher rates of severe COVID-19 outcomes, including hospitalisation and mortality, among men.^1^ While infection rates were elevated among working-age women compared with those younger or retired,^2 3^ women, in contrast to men, appeared relatively protected from adverse health consequences, potentially due to differing biological responses.^4 5^

Aside from biological factors, socioeconomic and behavioural characteristics probably contributed to the unequal distribution of severe COVID-19 outcomes between women and men, with occupation and workplace settings playing crucial roles in exposure and transmission.^6^ Key occupational factors influencing exposure include contact frequency, physical proximity, work environment and access to protective equipment.^7^ Women often occupy contact-intensive roles, notably in the service sector, such as healthcare, food preparation and education,^8^ where essential and/or frontline occupations are overrepresented.^9^ Men are more prevalent in less contact-intensive roles in the construction and manufacturing sectors.^8^ Workplace contacts were linked to increased COVID-19 infection risks in healthcare and trade, process and plant professions, often operating in poorly ventilated workplaces, emphasising the occupational influence on viral exposure.^10^

Certain occupational groups faced not only higher exposure risks but also increased adverse outcomes. Studies in the UK found elevated COVID-19 mortality risks among male taxi drivers and female healthcare professionals,^11 12^ although risks in the latter group attenuated after the first pandemic wave.^13^ Similar results were reported for Scotland alone, indicating higher risks of hospitalisation and severe COVID-19 among healthcare workers and their households.^14^

Behavioural factors also contribute to occupational and sex/gender disparities in COVID-19 outcomes, with women displaying greater compliance with preventing measures, including hygiene practices, mask wearing and social distancing.^15^ Conversely, men tend to engage in high-risk and health-damaging behaviours in general,^16^ along with lower healthcare-seeking behaviours, both of which potentially result in worse outcomes in relation to COVID-19.^17^

Moreover, household circumstances and pre-existing health conditions intersect with occupational disparities, with low-paid, low-skilled workers facing higher exposure risks due to overcrowded accommodation, higher propensity to coreside with younger adults or children and higher levels of comorbidities.^17^ Conditions like cardiovascular disease, diabetes and respiratory problems heighten COVID-19 mortality risks,^18^ particularly among older adults from ethnic minority groups in multigenerational households.^19^

In this study, we leveraged a large Scottish data collection covering the period between 1 March 2020 and 31 January 2021. Using Cox proportional hazards models, we addressed the following research questions: (1) Does the association between occupation and COVID-19 hospital admission and mortality risks differ by sex? (2) Is this association explained by pre-pandemic health and occupational exposure factors?

## Methods

### Data and study population

We used a large data collection covering the Scottish population, combining the following:

1. Socioeconomic information from 2011 Census.
2. Residential address identifiers from Ordnance Survey.
3. Occupational exposure measures from the Occupational Information Network (O*NET) survey.
4. Electronic health information on mortality, hospitalisation, laboratory testing and primary care records from Public Health Scotland’s (PHS) COVID-19 Research Database.^20^

The data collection is part of a wider project investigating social risk factors for COVID-19 in Scotland,^21^ originally established within the Scottish Data and Intelligence Network^22^ to inform the government’s response to the pandemic and now focused on long-term lessons.

**Figure 1:**
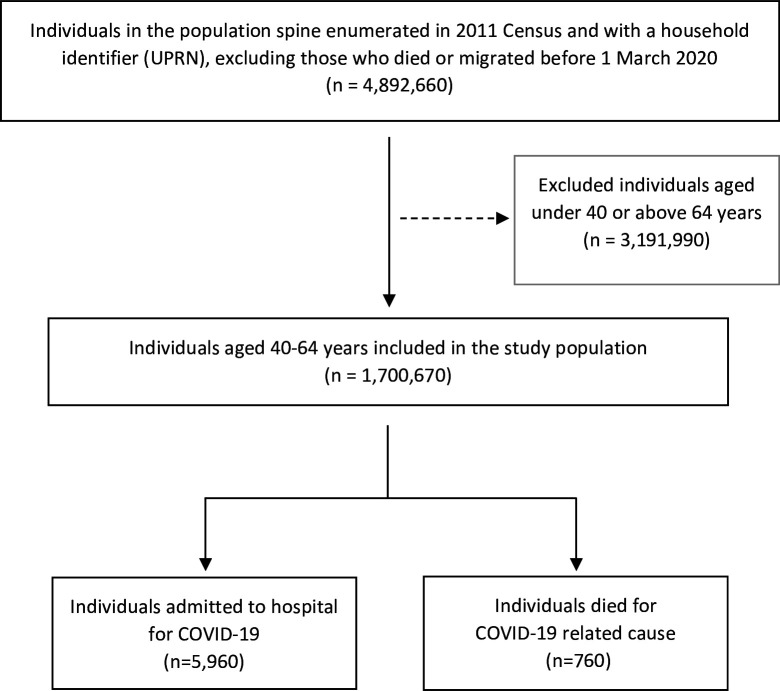
Flowchart representing the selection process for the study population. Reported figures are rounded to the nearest 10; UPRN, Unique Property Reference Number.

The Scottish population spine encompasses all individuals registered with a GP practice, who received a unique Community Health Index (CHI) number. Our study focused on individuals present in the Scottish 2011 Census with records linkable to the population spine. The Census provided occupation information, which we used as a measure of likely occupation in 2020/2021. We excluded those who died or relocated from Scotland before 1 March 2020 (the date of the first COVID-19 case in Scotland). The population spine provided the latest address recorded by the General Practice. Address information was used to attach a household identifier, derived from Ordnance Survey’s Unique Property Reference Numbers (UPRNs), through an innovative residential linkage tool.^23^ Following subsequent linkage to electronic health records, data were available for 4.9 million people, representing approximately 90% of the Scottish population.^24^ The study population comprised individuals aged 40–64 years on 1 March 2020, resulting in a cohort of approximately 1.7 million individuals after exclusions (figure 1).

### Outcomes

The outcomes of interest were time to first COVID-19 hospital admission and death. Hospital admission was identified using International Classification of Diseases 10th edition (ICD-10) codes for confirmed (U07.1) or suspected COVID-19 (U07.2) as primary or secondary causes, or admissions occurring within 14 days of a laboratory-confirmed COVID-19 infection. Death from COVID-19 was defined using ICD-10 codes for confirmed or suspected COVID-19 (primary or secondary cause) reported on death certificates and identified within 28 days of a laboratory-confirmed COVID-19 infection. These definitions align with those used in Scottish official statistics.^25^

### Exposure

We constructed an occupational measure using the UK Standard Occupational Classification (SOC) 2010 reported in the 2011 Census,^26^ acknowledging that SOC classifications have changed over time. Based on SOC 2010, we identified 26 occupational groups ranging from major groups (1-digit codes) to more detailed unit groups (4-digit codes) (online supplemental table S1). This combination of occupational groups was selected to identify high-risk occupations related to COVID-19 that were of particular policy relevance. Where sufficient sample sizes were available, we identified groups such as taxi drivers and health professionals, as these were of particular interest based on prior studies,^11^,^14^ with which we aimed to maintain comparability. Occupational groups with smaller sample sizes or lower policy relevance were aggregated to SOC codes of three digits or fewer. Groups were always exclusive. For example, professional occupations (SOC code: 2) included all lower level subgroups except for health professionals (SOC code: 22) and business, media and public service professionals (SOC code: 24) as these were included separately.

Some discrepancies may arise between the occupation reported in the 2011 Census and actual occupation in March 2020. Since both add *noise* to our measure, estimates of the relationships between occupation and COVID-19 risks should be regarded as lower limits. Note that individuals may have experienced job displacement during the COVID-19 pandemic; previous research indicated women experienced greater job losses than men.^8^ Using UK Household Longitudinal Study (UKHLS) data,^27^ we computed the proportion of women and men aged 40–64 years who retained the same occupation between 2011 and 2020 using major group occupations. For women, proportions varied between 40.8% among managers, directors and senior officials and 74.1% among workers in caring, leisure and other service occupations. For men, proportions varied between 38.8% among those in sales and customer service occupations and 72% among process, plant and machine operatives (online supplemental table S2). Despite limited disaggregation, these results suggest that occupational information in 2011 Census was relatively stable over time.

### Covariates

Covariates encompassed factors potentially associated with both occupation and COVID-19 hospital admission and mortality risks (online supplemental table S3). Sociodemographic factors included age, sex (from CHI register) and ethnicity (2011 Census). Household-level factors included housing tenure (2011 Census), household size, presence of children and multigenerational household based on people’s age cohort (from UPRN-based household identifiers). Health-related factors included learning disability or learning difficulty (2011 Census) and shielding status due to underlying vulnerable conditions (PHS shielding patient list dataset) (online supplemental table S4). Shielding status was measured based on receipt of a National Health Service (NHS) letter asking individuals to shield, reflecting NHS selection criteria rather than individual behaviour. Pre-pandemic health conditions were derived from cluster variables based on Read codes recorded in primary care data (PHS COVID-19 Research Database). Cancer and immunosuppression, cardiovascular conditions, diabetes, hypertension, respiratory conditions and other conditions were considered factors likely to heighten severe COVID-19 risks^18 28^ (online supplemental table S5). Occupational exposure measures (frequency of exposure to disease/infection, physical proximity to others and frequency of working in environmentally-controlled indoor settings) were sourced from US survey data from O*NET^29^ and mapped onto UK SOC 2010 codes, via ISCO-08 codes, and standardised using Office for National Statistics procedures.^30^

### Statistical analysis

We summarised the individual-level and household-level characteristics for the total population and for those who were first admitted to hospital and died of COVID-19. We reported baseline characteristics for covariates of interest using counts and percentages. Age-standardised COVID-19 hospital admission and mortality rates per 100 000 persons were calculated for each occupational group and annualised. Rates were estimated separately for women and men, using a direct method and the 2013 European Standard Population.^31^ CIs were calculated using Dobson *et al*’s method^32^ accounting for small number of events. Estimates were produced using the ‘distrate’ command^33 34^ in Stata/MP V.16 (StataCorp LP, College Station, Texas).

We modelled the time to COVID-19 hospital admission and death using Cox proportional hazards models. Analyses were conducted both through separate models for women and men, and through a combined model including an interaction between occupation and sex. While the interaction model helps determine whether interaction parameters are statistically significant, we relied on the separate models to estimate the magnitude of the association between occupation and COVID-19 outcomes within each sex group. We estimated a series of models, sequentially adjusting for potential confounders and mediators. Models 1–4 are estimated for each sex separately. Model 5 is estimated for both sexes together. Model 1 adjusted for sociodemographic factors, such as age and ethnicity, with occupation as the main exposure. A restricted cubic spline was used to model the non-linear association between age and COVID-19 outcomes. Model 2 additionally adjusted for household-level factors, including housing tenure, household size and composition, and whether the household was multigenerational. Model 3 additionally accounted for health-related factors, including learning disability/difficulty, shielding status and pre-pandemic health conditions. Model 4 added adjustments for occupational exposure measures, including exposure to disease, proximity to others and environmentally-controlled indoor conditions. This is referred to as the ‘fully adjusted model’. Model 5 has all the adjustments of Model 4 but uses data for both sexes and includes an interaction term between occupation and sex. The hypothesised relationships between the variables included in the models are illustrated in a causal diagram (online supplemental figure S1). We acknowledge that it is unclear from the available data whether some of the factors may act as confounders or mediators. Model fit was assessed using Akaike Information Criterion (AIC) and Bayesian Information Criterion (BIC) statistics and Likelihood Ratio (LR) tests. Analyses were conducted in the Scottish National Safe Haven using Stata/MP V.16 and R (V.3.6.1).

## Results

The study encompassed 1 700 670 Scottish adults aged 40–64 years, observed between 1 March 2020 and 31 January 2021. The mean age at baseline was 52.4 years (SD 7.0), with 50.6% being women. Individuals were followed for 1 514 189 person-years until first COVID-19 hospital admission, 1 561 010 person-years until COVID-19 death or until the end of the follow-up period on 31 January 2021. Among 5960 COVID-19 admissions, the median follow-up was 232 days (95% CI 228 to 233), with admission events more likely to occur among men (52.7%) and older individuals (mean age 54.7 years (SD 6.4)). Among the 760 COVID-19 deaths, the median follow-up was 248 days (95% CI 237 to 253), with 460 deaths (60.5%) occurring among men and a mean age of 57.2 years (SD 5.8). Additional baseline characteristics are detailed in table 1.

**Table 1:** Characteristics of the study population and those who were admitted to hospital or died for a COVID-19 cause

Characteristics	Population* (n=1 700 670)		COVID-19 hospital admissions* (n=5960)		COVID-19 deaths* (n=760)	
	N	%	n	%	n	%
Sex						
Women	859 800	50.56	2820	47.32	300	39.47
Men	840 870	49.44	3140	52.68	460	60.53
Age (years)†	52.40	7.00	54.70	6.40	57.20	5.80
Ethnicity						
Non-white	195 810	11.51	770	12.92	90	11.84
White	1 504 860	88.49	5190	87.08	670	88.16
Occupation (SOC 2010 codes)						
1 - Managers, directors and senior officials	136 730	8.04	460	7.72	50	6.58
2 - Professional occupations	118 250	6.95	190	3.19	20	2.63
22 - Health professionals	74 150	4.36	310	5.20	10	1.32
24 - Business, media and public service professionals	65 340	3.84	120	2.01	10	1.32
3 - Associate professional and technical occupations	76 370	4.49	190	3.19	20	2.63
33 - Protective service occupations	26 910	1.58	100	1.68	10	1.32
35 - Business and public service associate professionals	88 630	5.21	170	2.85	20	2.63
41 - Administrative occupations	138 500	8.14	390	6.54	50	6.58
42 - Secretarial and related occupations	40 180	2.36	120	2.01	10	1.32
5 - Skilled trades occupations	90 400	5.32	290	4.87	30	3.95
53 - Skilled construction and building trades	60 580	3.56	220	3.69	30	3.95
543 - Food preparation and hospitality trades	29 060	1.71	110	1.85	20	2.63
6 - Caring, leisure and other service occupations	70 140	4.12	230	3.86	20	2.63
614 - Caring personal services	28 470	1.67	180	3.02	10	1.32
6145 - Care workers and home workers	52 310	3.08	300	5.03	30	3.95
71 - Sales occupations	87 000	5.12	320	5.37	50	6.58
72 - Customer service occupations	26 820	1.58	120	2.01	10	1.32
81 - Process, plant and machine operatives	41 230	2.42	180	3.02	30	3.95
811 - Process plant operatives	19 940	1.17	90	1.51	20	2.63
82 - Transport and mobile machine drivers and operatives	40 830	2.40	210	3.52	30	3.95
8211 - Large goods vehicle drivers	17 040	1.00	90	1.51	20	2.63
8214 - Taxi and cab drivers and chauffeurs	12 170	0.72	110	1.85	20	2.63
9 - Elementary occupations	59 540	3.50	240	4.03	40	5.26
91 - Elementary trades and related occupations	34 600	2.03	130	2.18	30	3.95
927 - Other elementary service occupations	38 440	2.26	160	2.68	20	2.63
9233 - Cleaners and domestics	41 680	2.45	200	3.36	30	3.95
No code required	185 360	10.90	730	12.25	120	15.79
Housing tenure						
Owned outright	226 160	13.30	600	10.07	80	10.53
Owned with mortgage	897 530	52.78	2960	49.66	300	39.47
Social rented	282 060	16.59	1520	25.50	260	34.21
Private rented	138 700	8.16	380	6.38	50	6.58
Owned/not known	156 220	9.19	500	8.39	70	9.21
Household size						
1–2 people	779 040	45.81	2860	47.99	420	55.26
3–4 people	720 230	42.35	2310	38.76	220	28.95
5–6 people	160 170	9.42	540	9.06	40	5.26
7+people	41 230	2.42	250	4.19	80	10.53
Household with children						
No children	1 300 090	76.45	4920	82.55	690	90.79
At least one child 0–11 years	187 120	11.00	440	7.38	30	3.95
At least one child 12–17 years	213 460	12.55	600	10.07	40	5.26
Whether multigenerational household						
Yes	141 970	8.35	530	8.89	640	84.21
No	1 558 700	91.65	5430	91.11	120	15.79
Occupational exposure measures†						
Exposure to disease	0.30	0.30	0.30	0.30	0.30	0.30
Proximity to others	0.60	0.20	0.60	0.30	0.50	0.30
Environmentally controlled indoor conditions	0.70	0.30	0.60	0.30	0.60	0.30
Learning disability or difficulty						
Yes	31 770	1.87	210	3.52	50	6.58
No	1 518 930	89.31	5310	89.09	660	86.84
Not known	149 970	8.82	440	7.38	50	6.58
Whether shielding						
Yes	39 100	2.30	630	10.57	150	19.74
No	1 661 570	97.70	5330	89.43	610	80.26
Pre-pandemic health conditions						
Cancer and immunosuppression						
Yes	23 960	1.41	190	3.19	30	3.95
No	1 676 710	98.59	5770	96.81	730	96.05
Cardiovascular conditions						
Yes	118 880	6.99	960	16.11	190	25.00
No	1 581 790	93.01	5000	83.89	570	75.00
Diabetes						
Yes	104 150	6.12	1130	18.96	200	26.32
No	1 596 520	93.88	4830	81.04	560	73.68
Hypertension						
Yes	241 210	14.18	1590	26.68	230	30.26
No	1 459 460	85.82	4370	73.32	530	69.74
Respiratory conditions						
Yes	246 490	14.49	1420	23.83	210	27.63
No	1 454 180	85.51	4540	76.17	550	72.37
Other conditions						
Yes	308 710	18.15	1650	27.68	270	35.53
No	1 391 960	81.85	4310	72.32	490	64.47

* Hospital admissions and deaths occurring between 1 March 2020 and 31 January 2021; reported figures are rounded to the nearest 10.

† Mean and SD are reported.

Table 2 shows annualised age-standardised COVID-19 hospital admission and death rates for women and men aged 40–64 years. In general, women had lower age-standardised rates (ASRs) than men across both outcomes. For women, the highest ASRs for COVID-19 admissions were among those in caring personal services, including nursing assistants and ambulance staff (excluding paramedics), with 599.7 admissions (95% CI 499.0 to 714.1) per 100 000 persons, and process, plant and machine operatives (assemblers and sorters in the food and tobacco industry), with 576.8 admissions (95% CI 426.9 to 645.0). Men exhibited the highest ASRs for COVID-19 admissions among taxi and cab drivers (949.8 admissions per 100 000 persons, 95% CI 763.8 to 1164.6), workers in caring personal services (916.2 admissions per 100 000 persons, 95% CI 663.0 to 1231.7) and care workers and home workers (736.1 admissions per 100 000 persons, 95% CI 553.3 to 959.2). Professionals in science, research, engineering and technology as well as teaching and education reported the lowest (or second-lowest) rates for both women (150.9 admissions, 95% CI 120.4 to 186.8) and men (190.4 admissions, 95% CI 155.7 to 230.6).

**Table 2:** Annualised age-standardised rates (ASR) for COVID-19 hospital admissions and deaths per 100 000 persons aged 40–64 years in Scotland, by occupation and sex

SOC2010	Occupation	COVID-19 hospital admissions*		COVID-19 deaths*	
		Women (n=2820)	Men (n=3140)	Women (n=300)	Men (n=460)
Code		ASR (95% CI)†	ASR (95% CI)†	ASR (95% CI)†	ASR (95% CI)†
1	Managers, directors and senior officials	255.6 (214.2 to 302.6)	382.6 (340.5 to 428.3)	29.4 (16.8 to 47.9)	40.8 (28.2 to 57.1)
2	Professional occupations	150.9 (120.4 to 186.8)	190.4 (155.7 to 230.6)	15.4 (7.0 to 29.3)	21.1 (10.9 to 36.8)
22	Health professionals	433.9 (380.8 to 492.2)	511.6 (392.8 to 654.7)	19.5 (9.7 to 34.9)	8.8 (0.2 to 48.8)
24	Business, media and public service professionals	130.8 (91.0 to 182.2)	238.0 (188.6 to 296.2)	–	28.4 (13.6 to 52.4)
3	Associate professional and technical occupations	218.6 (172.6 to 272.9)	325.0 (268.7 to 389.5)	10.5 (2.9 to 26.9)	43.7 (25.0 to 71.0)
33	Protective service occupations	–	457.6 (368.3 to 561.8)	–	51.6 (25.4 to 92.9)
35	Business and public service associate professionals	161.9 (123.9 to 207.9)	252.2 (206.9 to 304.4)	7.9 (1.6 to 23.1)	35.9 (20.5 to 58.4)
41	Administrative occupations	277.8 (246.5 to 311.9)	371.4 (302.4 to 451.4)	30.2 (20.7 to 42.5)	62.6 (37.0 to 99.1)
42	Secretarial and related occupations	303.0 (247.8 to 366.4)	–	29.5 (14.7 to 52.4)	–
5	Skilled trades occupations	226.1 (127.2 to 368.6)	327.1 (288.7 to 369.2)	34.8 (1.8 to 132.8)	33.0 (22.1 to 47.4)
53	Skilled construction and building trades	–	369.8 (322.0 to 422.6)	–	52.4 (35.7 to 74.1)
543	Food preparation and hospitality trades	346.2 (251.9 to 463.7)	451.7 (349.8 to 573.9)	28.0 (7.6 to 71.7)	76.4 (38.0 to 137.0)
6	Caring, leisure and other service occupations	327.7 (281.8 to 378.9)	472.9 (345.9 to 630.5)	18.8 (8.9 to 34.7)	64.5 (22.7 to 142.4)
614	Caring personal services	599.7 (499.0 to 714.1)	916.2 (663.0 to 1231.7)	42.4 (19.1 to 79.9)	45.1 (3.9 to 167.6)
6145	Care workers and home workers	566.4 (494.9 to 645.0)	736.1 (553.3 to 959.2)	60.5 (39.6 to 88.3)	59.9 (19.5 to 139.8)
71	Sales occupations	365.3 (320.3 to 414.8)	477.6 (378.9 to 593.9)	50.9 (35.4 to 70.8)	84.8 (46.2 to 142.4)
72	Customer service occupations	499.9 (398.6 to 619.0)	457.8 (315.9 to 641.1)	41.3 (16.6 to 85.2)	54.3 (14.7 to 139.4)
81	Process, plant and machine operatives	576.8 (426.9 to 759.7)	392.2 (324.7 to 469.4)	108.6 (57.6 to 186.2)	54.4 (31.4 to 87.6)
811	Process plant operatives	440.3 (299.2 to 622.6)	411.6 (305.6 to 542.3)	40.9 (11.1 to 104.9)	84.4 (42.1 to 151.2)
82	Transport and mobile machine drivers and operatives	336.3 (159.5 to 621.4)	498.4 (428.8 to 575.9)	31.3 (0.8 to 174.2)	60.8 (38.8 to 90.5)
8211	Large goods vehicle drivers	–	501.2 (392.5 to 628.9)	–	95.7 (56.2 to 151.9)
8214	Taxi and cab drivers and chauffeurs	–	949.8 (763.8 to 1164.6)	–	132.9 (79.4 to 208.5)
9	Elementary occupations	310.7 (225.4 to 416.7)	441.5 (381.1 to 508.6)	77.0 (39.3 to 135.3)	64.2 (42.9 to 92.3)
91	Elementary trades and related occupations	418.8 (282.1 to 596.9)	377.2 (306.4 to 459.3)	127.6 (55.6 to 246.7)	67.2 (39.7 to 106.3)
927	Other elementary service occupations	403.3 (331.9 to 485.5)	605.7 (447.6 to 801.4)	33.5 (16.1 to 61.7)	149.0 (76.8 to 260.4)
9233	Cleaners and domestics	452.4 (385.8 to 526.9)	342.9 (205.2 to 537.2)	56.9 (36.3 to 85.0)	74.1 (19.7 to 190.9)

* Hospital admissions and deaths occurring between 1 March 2020 and 31 January 2021.

† 95% CIs calculated using the Dobson method^32^ are reported; ASRs are standardised to the 2013 European Standard population using the direct method, and annualised; estimates are not reported if there were less than 10 admissions or deaths.

SOC 2010, Standard Occupational Classification 2010.

COVID-19 mortality ASRs displayed a steeper occupational gradient compared with hospital admission ASRs. Among women, the highest ASR was among those in elementary trades and related occupations, such as packers and canners (127.6 COVID-19 deaths per 100 000 persons, 95% CI 55.6 to 246.7), while business and public service associate professionals, including those in the transport and legal administration sectors, had the lowest ASR (7.9 deaths per 100 000 persons, 95% CI 1.6 to 23.1). For men, the highest ASR was among those in other elementary service occupations, like kitchen assistants and waiters (149 deaths per 100 000 persons, 95% CI 76.8 to 260.4), while professionals had one of the lowest ASRs (21.1 deaths per 100 000 persons, 95% CI 10.9 to 36.8).

**Figure 2:**
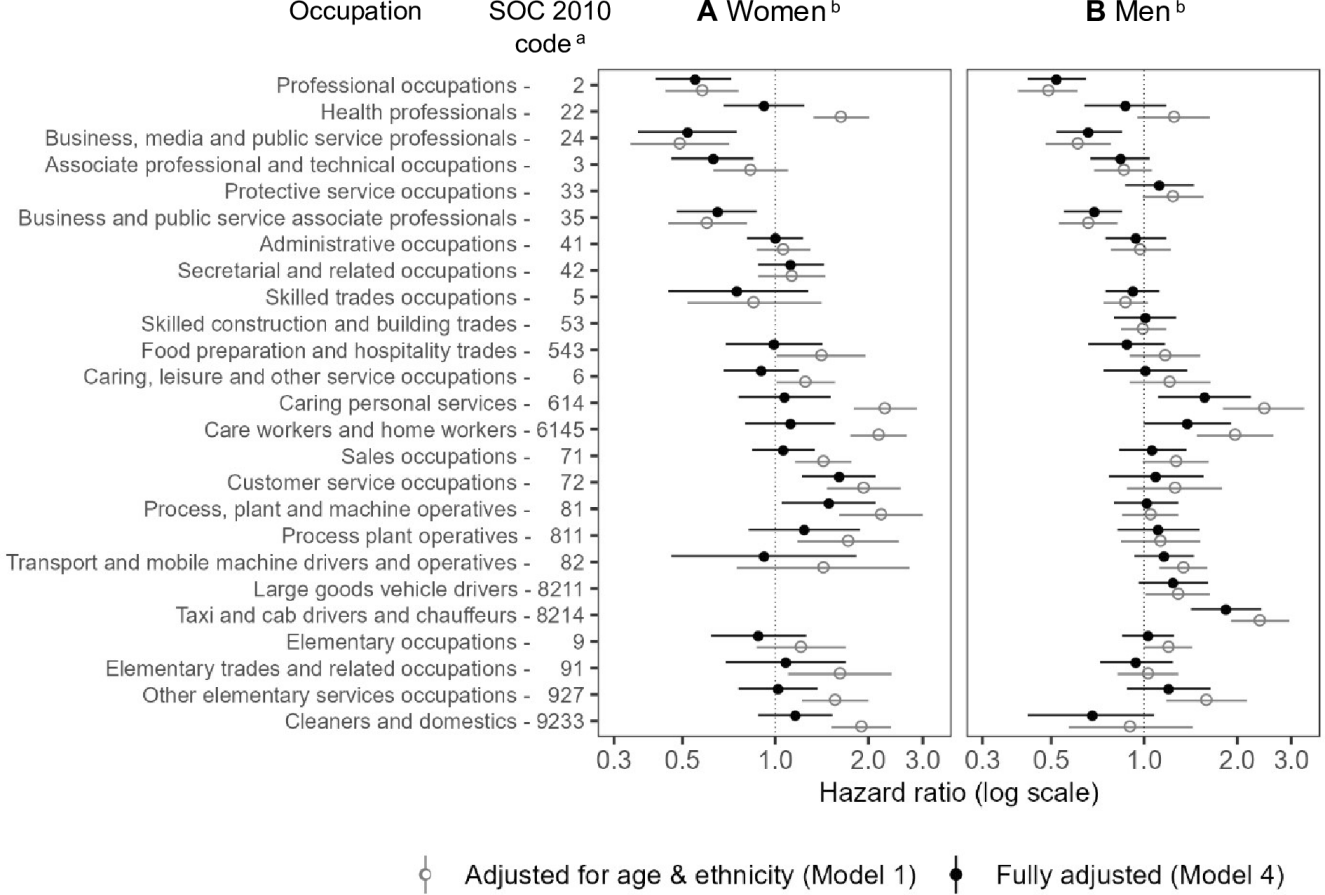
Hazard ratios for COVID-19 hospital admission for (**A**) women and (**B**) men aged 40–64 years in Scotland. ^a^The Standard Occupational Classification 2010 (SOC2010) is a nested classification. Where a higher-level code is indicated, this includes all lower-level subgroups except for the specific subgroups that are separately identified (eg, professional occupations (SOC code: 2) includes all lower-level subgroups except for health professionals (SOC code: 22) and business, media and public service professionals (SOC code: 24). ^b^Hazard ratios and 95% CIs are displayed on a logarithmic scale; Reference category: SOC code 1: managers, directors and senior officials; fully-adjusted model (Model 4) is coloured in black (solid circle) and appears above Model 1 (grey hollow circle) for each group; fully-adjusted model (Model 4, online supplemental table S6) additionally controls for: household-level factors (housing tenure, household size, household with children, whether multigenerational household), health-related factors (learning disability or difficulty, whether shielding and pre-pandemic health conditions), and occupational exposure measures (exposure to disease, proximity to others, environmentally-controlled indoor conditions); hospital admissions occurring between 1 March 2020 and 31 January 2021; data are not reported if there were less than 10 hospital admission events.

The relative difference in COVID-19 hospital admission risks between the occupational groups and managers, directors and senior officials (reference category) decreased after adjusting for confounding factors (figure 2; online supplemental tables S6 and S8 for full-model and interaction model details, respectively). However, for some occupational groups, risks remained significantly higher relative to the reference category. For example, in the fully-adjusted model, women in customer service occupations had a HR of 1.61 (95%CI 1.22 to 2.11) compared with 1.93 (95%CI 1.47 to 2.54) in the baseline model adjusting for age and ethnicity. An elevated risk also remained for process, plant and machine operatives (assemblers and sorters) (HR 1.49, 95% CI 1.05 to 2.11). Among men, elevated risks persisted for taxi and cab drivers (HR 1.84, 95% CI 1.42 to 2.40), those working in caring personal services (ambulance staff) (HR 1.57, 95% CI 1.11 to 2.22) and care and home workers (HR 1.38, 95% CI 1.00 to 1.91).

Lower COVID-19 admission risks were observed among less disadvantaged occupational groups after adjusting for household characteristics and pre-pandemic health conditions, for both women and men. For instance, professionals had an HR of 0.55 (95% CI 0.41 to 0.72) for women and 0.52 (95% CI 0.42 to 0.65) for men, compared with 0.58 (95% CI 0.44 to 0.76) and 0.49 (95% CI 0.39 to 0.61), respectively, in the baseline model. Similarly, professionals in business, media and public service had HRs of 0.52 (95% CI 0.36 to 0.75) for women and 0.66 (95% CI 0.52 to 0.85) for men, compared with 0.65 (95% CI 0.48 to 0.87) and 0.69 (95% CI 0.55 to 0.85), respectively.

**Figure 3:**
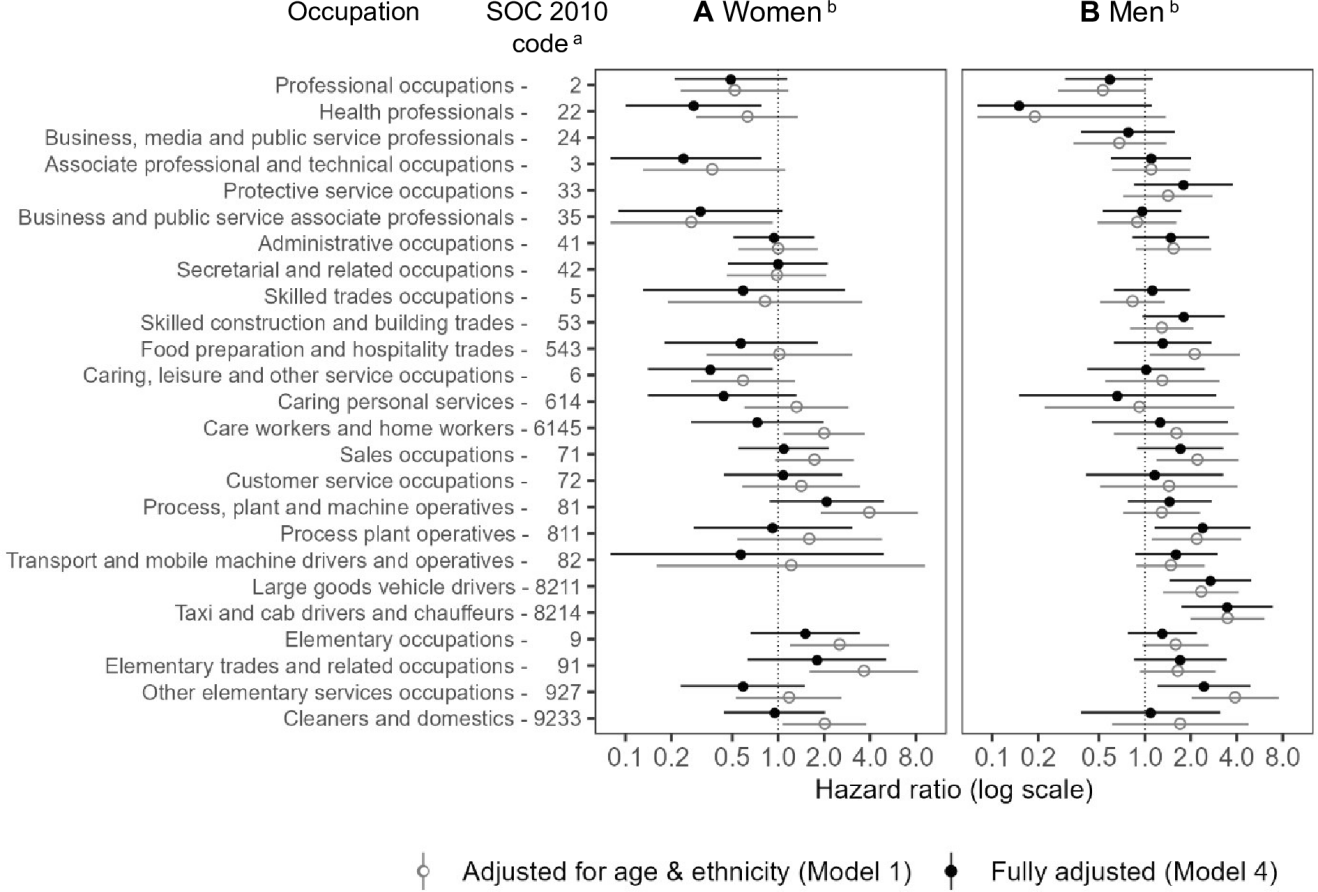
Hazard ratios for COVID-19 death for (**A**) women and (**B**) men aged 40–64 years in Scotland. ^a^The Standard Occupational Classification 2010 (SOC2010) is a nested classification. Where a higher-level code is indicated, this includes all lower-level subgroups except for the specific subgroups that are separately identified (eg, professional occupations (SOC code: 2) includes all lower-level subgroups except for health professionals (SOC code: 22) and business, media and public service professionals (SOC code: 24). ^b^Hazard ratios and 95% CIs are displayed on a logarithmic scale; reference category: SOC code 1: managers, directors and senior officials; fully-adjusted model (Model 4) is coloured in black (solid circle) and appears above Model 1 (grey hollow circle) for each group; fully-adjusted model (Model 4, online supplemental table S7) additionally controls for: household-level factors (housing tenure, household size, household with children, whether multigenerational household), health-related factors (learning disability or difficulty, whether shielding and pre-pandemic health conditions) and occupational exposure measures (exposure to disease, proximity to others, environmentally-controlled indoor conditions); deaths occurring between 1 March 2020 and 31 January 2021; data are not reported if there were less than 10 deaths.

Differences between women and men emerged regarding the risk of COVID-19 death by occupation (figure 3; online supplemental tables S7 and S8 for full model and interaction model details, respectively). Female health professionals (medical practitioners, nurses and pharmacists) had a lower death risk relative to the reference group (HR 0.28, 95% CI 0.10 to 0.78), as did women in associate professional and technical occupations (HR 0.24, 95% CI 0.07 to 0.78), for whom a significant difference relative to men was also observed in the interaction model (HR 0.23, 95% CI 0.08 to 0.70). Lower death risks relative to the reference group were observed for women among health and social care workers (HR 0.24, 95% CI 0.07 to 0.78), and in caring, leisure and other service occupations (childminders and nursery assistants) (HR 0.36, 95% CI 0.14 to 0.92). Conversely, men working as taxi and cab drivers (HR of 3.46, 95% CI 1.74 to 6.86) and large goods vehicle drivers (HR 2.69, 95% CI 1.45 to 4.99) exhibited increased COVID-19 death after adjusting for socioeconomic and health factors. Elevated risks were also shown for those in other elementary service occupations, including kitchen assistants and waiters (HR 2.44, 95% CI 1.21 to 4.91) and process plant operatives (HR 2.39, 95% CI 1.16 to 4.91).

## Discussion

### Main results

Drawing on a large data collection, using Census, electronic health and residential data, we estimated COVID-19 hospital admission and mortality risks for different occupational groups in Scotland, focussing on women and men aged 40–64 years during the period between March 2020 and January 2021, predating the vaccination programme. Overall, women exhibited lower age-standardised COVID-19 admission and mortality rates. For both women and men, rates were largely consistent with an occupational gradient, with higher rates among more disadvantaged occupational groups, related to both COVID-19 admission and death risks. Notably, women in process, plant and machine operative roles (assemblers and sorters), along with elementary trades and related occupations (packers and canners) had the highest age-standardised mortality rates. Similarly, women in caring personal services (nursing assistants and ambulance staff) had elevated admission rates. Conversely, men in elementary services (kitchen assistants and waiters) and transportation roles, notably taxi and cab drivers, had the highest age-standardised mortality rates, with the latter group also having high admission rates. Similar trends were observed in previous studies, where higher mortality rates were found among women in process, plant and machine operative roles, while taxi and cab drivers were among the categories with the highest mortality rates among men.^11 35^

Adjusting for socioeconomic, pre-pandemic health and occupational exposure factors reduced the association between COVID-19 hospital admission and death risks for most occupational groups, compared with the baseline model adjusting for basic demographic factors. After adjustment, we generally observed a similar occupational gradient for COVID-19 admission risks for women and men. For COVID-19 mortality risks, an occupational gradient also persisted after adjustment showing lower risks among women in less disadvantaged occupational groups (health professionals, associate professional and technical occupations, including paramedics and medical technicians, with the latter supported by the interaction model results) and higher risks among men in more disadvantaged occupational groups (taxi and large vehicle drivers, other elementary service occupations, including kitchen assistants and waiters, and process plant operatives in the food, drink and tobacco industry). Although these results generally align with previous studies, our study contrasts with one from England, which observed elevated mortality risks among men in health professional occupations but not among women.^11^ In population-based studies that did not differentiate between women and men, healthcare professionals and associates showed higher proportionate mortality odds only during the first wave of the pandemic, but not in subsequent waves in England and Wales.^13^ Additionally, increased severe COVID-19 risks were reported among healthcare workers, relative to non-essential workers, in the general UK population.^12^ These discrepancies may reflect differences in access to protective equipment or adherence to preventive measures.

While household characteristics and pre-pandemic health and occupational exposure factors were important in our study in explaining the association between COVID-19 admission and death risks for women employed in the service and industrial sectors (care worker and home workers, and process, plant and machine operatives), they did not explain the elevated risks for men in more disadvantaged occupations. An alternative explanation could include a combination of behavioural factors (eg, compliance with guidance) and workplace-related factors (eg, contact density, ventilation, remote working and financial strain).

### Strengths

To our knowledge, this is the first Scottish study to estimate COVID-19 hospital admission and mortality risks across occupational groups among women and men, adjusting for a range of confounders. First, covering a population of 1.7 million, our data collection was well-powered to assess the outcome of interest across occupational groups by sex. Our results are likely to be relevant for other parts of the UK and other countries. Previous studies examined these associations in national or subpopulations,^12 14 35^ while others reported only COVID-19 mortality risks.^11 36^ Second, leveraging Unique Property Reference Numbers (UPRNs), we derived a set of household-level covariates, improving on 2011 Census-based measures used in prior studies.^11 19^ Third, in our analysis, we adjusted for both pre-pandemic health measures—derived by combining Census and primary care records—and occupational exposure measures, including exposure to disease, proximity to other and environmentally-controlled indoor conditions. Adjusting for pre-pandemic health and occupational exposure factors was overlooked in previous research.^12 13 35 37 38^

### Limitations

A limitation of this study lies in potential discrepancies from using 2011 Census occupational information, as people may not be employed in the same occupation in March 2020 and there may be subsequent changes during the pandemic. While this adds noise to our measure, so that estimates of the relationships between occupation and COVID-19 risks should be considered as lower bounds, we believe the bias is likely to be toward the null. As a mitigation, we focused on a less occupationally mobile age group, that is, those aged 40–64 years^11^. Corroborating this, we used UKHLS data showing relatively high proportions of men and women retaining the same occupation between 2011 and 2020, suggesting that the Census occupation information was sufficiently stable over time. Our study estimates are largely consistent with both official and other estimates from population-based studies using linked administrative data.^1113 35^,^37^ The occupational classification approach used in our study differs from previous investigations using broader essential work clusters,^13 37^ as we think that women and men may differ in the position and roles within the same occupational cluster.

Another limitation is the potential bias in the way outcomes were ascertained. During the pandemic, certain occupations (eg, patient-facing or public-facing roles) were tested more frequently or had a higher likelihood of being tested due to the nature of their work. This may have influenced the outcomes related to hospital admissions within 14 days of a COVID-19 infection and suspected COVID-19 cases as well as subjective judgements regarding the cause of death reported on death certificates.

An additional limitation concerns the data linkage design. Our initial study population covers approximately 90% of the Scottish population. The data linkage relies on a population spine covering people who interacted with the health services, receiving a CHI number. The population spine may not cover some vulnerable groups (eg, refugees and migrants) that may be at increased risks of severe COVID-19. Potential bias may arise from the linkage being conditional on having a valid 2011 Census record or a matched key for a UPRN residential identifier.^23^ Misclassification may also arise due to errors in linked data and the data linkage process.^39^ We acknowledge these sources of bias, although their impact on estimates remains uncertain. Additionally, the relationship between occupation and COVID-19 outcomes has changed over the course of the pandemic.^13 40^ However, rare outcomes have limited the exploration of time-dependent differences as well as the evaluation of changing restriction policies across pandemic waves. Moreover, limitations in statistical power and precision may have affected the confidence intervals of the parameter estimates. In addition, some of our variables could theoretically act as either confounding or mediating factors. However, because they are only measured at a single point in time, it is not possible to determine their causal direction. As a result, caution is needed when interpreting the regression estimates. Future research should include additional background and workplace factors measured at multiple time points to support a formal mediation analysis and better assess the impact of occupation on COVID-19 outcomes attributable to pre-pandemic health and occupational exposure factors.

### Policy implications

Occupational differences in COVID-19 hospital admission and mortality risks between women and men may be explained by social, workplace and behavioural factors. These need to be considered when developing tailored interventions to reduce sex/gender discrepancies in any future emerging respiratory epidemics. Addressing gaps in protective equipment and training provision between health professionals and care and home workers is vital if we want to address inequalities arising from pandemic policy responses. Coordinated interventions targeting high-risk occupational groups, working in transportation and service sectors are imperative to mitigate transmission risks effectively.

## Supplementary material

10.1136/oemed-2024-109562 online supplemental file 1

## Data Availability

Data may be obtained from a third party and are not publicly available.
