# Supplementary material for: Occupational differences in COVID-19 hospital admission and mortality risks between women and men in Scotland: a population-based study using linked administrative data
Source: Occup Environ Med. 2025 Apr 29;82(3):e109562. doi: 10.1136/oemed-2024-109562 (PMC12171494; doi:10.1136/oemed-2024-109562)
Supplement: online supplemental file 1 [file oemed-82-3-s001.docx]

Supplementary material

Occupational differences in COVID-19 hospital admission and mortality risks between women and men in Scotland: a population-based study using linked administrative data

Table of Contents

[**Table S1** Occupational groups and SOC 2010 codes used in the study ^a^, including lower-level components (unit group, four-digit SOC 2010 codes) 2](#_Toc185068660)

[**Table S2** Proportion of adults aged 40-64 years remaining in the same occupation between 2011-13 and 2018-19 in the UK, by major group occupation and sex 4](#_Toc185068661)

[**Table S3** Covariates included in the statistical analysis 5](#_Toc185068662)

[**Table S4** Description of the groups of people and conditions included in the shielding patient list 6](#_Toc185068663)

[**Table S5** Clusters and Read codes used to derive pre-pandemic health conditions from primary care records 7](#_Toc185068664)

[**Figure S1** Directed Acyclic Graph (DAG) showing the hypothesised relationships between occupation, the outcome variables and other covariates included in the statistical analysis 9](#_Toc185068665)

[**Table S6** Full Cox proportional hazards models of risk of COVID-19 hospital admission for women and men aged 40-64 years in Scotland 10](#_Toc185068666)

[**Table S7** Full Cox proportional hazards models of risk of COVID-19 related death for women and men aged 40-64 years in Scotland 12](#_Toc185068667)

[**Table S8** Hazard ratios and confidence intervals for the interaction between occupation and sex in the prediction of COVID-19 hospital admission and COVID-19 death for adults aged 40-64 years in Scotland 14](#_Toc185068668)

# **Table S1** Occupational groups and SOC 2010 codes used in the study ^a^, including lower-level subgroups (unit groups with four-digit SOC codes)

| Occupational group ^a^ | SOC 2010 codes ^a^ | Lower-level subgroups  (unit groups with four-digit SOC codes) |
| --- | --- | --- |
|  |  |  |
| Managers, directors and senior officials | 1 | 1115, 1116, 1121-1123, 1131-1136, 1139, 1150, 1161, 1162, 1171-1173, 1181, 1184, 1190, 1211, 1213, 1221, 1223-1226, 1241, 1242, 1251-1255, 1259 |
|  |  |  |
| Professional occupations | 2 | 2111-2114, 2119, 2121-2124, 2126, 2127, 2129, 2133-2137, 2139, 2141, 2142, 2150, 2311, 2312, 2314-2319 |
|  |  |  |
| Health professionals | 22 | 2211-2219, 2221-2223, 2229, 2231, 2232 |
|  |  |  |
| Business, media and public service professionals | 24 | 2412, 2413, 2419, 2421, 2423-2426, 2429, 2431-2436, 2442-2444, 2449, 2451, 2452, 2461-2463, 2471-2473 |
|  |  |  |
| Associate professional and technical occupations | 3 | 3111-3116, 3119, 3121, 3122, 3131, 3132,3213, 3216-3219, 3231, 3233-3235, 3239, 3411-3417, 3421, 3422, 3441-3443 |
|  |  |  |
| Protective service occupations | 33 | 3311-3315, 3319 |
|  |  |  |
| Business and public service associate professionals | 35 | 3511-3513, 3520, 3531-3539, 3541-3546, 3550, 3561-3565, 3567 |
|  |  |  |
| Administrative occupations | 41 | 4112-4114, 4121-4124, 4129, 4131-4135, 4138, 4151, 4161, 4162 |
|  |  |  |
| Secretarial and related occupations | 42 | 4211-4217 |
|  |  |  |
| Skilled trades occupations | 5 | 5111-5114, 5119, 5211-5216, 5221-5225, 5231, 5232, 5234-5237, 5241,5242,5244, 5245, 5249, 5250, 5411-5414, 5419, 5421-5423, 5441-5443, 5449 |
|  |  |  |
| Skilled construction and building trades | 53 | 5311-5316, 5319, 5321-5323, 5330, |
|  |  |  |
| Food preparation and hospitality trades | 543 | 5431-5436 |
|  |  |  |
| Caring, leisure and other service occupations | 6 | 6121-6123, 6125, 6126, 6131, 6132, 6139, 6211, 6212, 6214, 6215, 6219, 6221, 6222, 6231, 6232, 6240 |
|  |  |  |
| Caring personal services | 614 | 6141-6144, 6146-6148 |
|  |  |  |
| Care workers and home workers | 6145 | 6145 |
|  |  |  |
| Sales occupations | 71 | 7111-7115, 7121-7125, 7129, 7130 |
|  |  |  |
| Customer service occupations | 72 | 7211, 7213-7215, 7219, 7220 |
|  |  |  |
| Process, plant and machine operatives | 81 | 8121-8127, 8129, 8131-8235, 8137, 8139, 8141-8143, 8149 |
|  |  |  |
| Process plant operatives | 811 | 8111-8119 |
|  |  |  |
| Transport and mobile machine drivers and operatives | 82 | 8212, 8213, 8215, 8221-8223, 8229, 8231-8234, 8239 |
|  |  |  |
| Large goods vehicle drivers | 8211 | 8211 |
|  |  |  |
| Taxi and cab drivers and chauffeurs | 8214 | 8214 |
|  |  |  |
| Elementary occupations | 9 | 9211, 9219, 9231-9236, 9239, 9241, 9242, 9249, 9251, 9259, 9260 |
|  |  |  |
| Elementary trades and related occupations | 91 | 9111, 9112, 9119, 9120, 9132, 9134, 9139 |
|  |  |  |
| Other elementary services occupations | 927 | 9271-9275, 9279 |
|  |  |  |
| Cleaners and domestics | 9244 | 9244 |

^a^ The Standard Occupational Classification 2010 (SOC2010) is a nested classification. Where a higher-level code is indicated, this includes all lower-level subgroups except for the specific subgroups that are separately identified (e.g. professional occupations (SOC code: 2) includes all lower-level subgroups except for health professionals (SOC code: 22) and business, media and public service professionals (SOC code: 24).

Source: Office for National Statistics. SOC2010 volume1: structure and descriptions of unit groups. 2010. <https://www.ons.gov.uk/methodology/classificationsandstandards/standardoccupationalclassificationsoc/soc2010/soc2010volume1structureanddescriptionsofunitgroups>. Accessed 15 August 2023.

# **Table S2** Proportion of adults aged 40-64 years remaining in the same occupation between 2011-13 and 2018-19 in the UK, by major group occupation and sex

| Major group occupation  (one-digit SOC 2010 code) | Population ^a^ | | | Women ^a^ | | | Men ^a^ | | |
| --- | --- | --- | --- | --- | --- | --- | --- | --- | --- |
|  | 2011-13  n | 2018-19  n | row  % ^b^ | 2011-13  n | 2018-19  n | row  % ^b^ | 2011-13  n | 2018-19  n | row  % ^b^ |
|  |  |  |  |  |  |  |  |  |  |
| 1 - Managers, directors and senior officials | 169 | 93 | 54.98 | 62 | 30 | 40.79 | 107 | 63 | 62.62 |
| 2 - Professional occupations | 290 | 202 | 70.91 | 152 | 111 | 73.18 | 138 | 91 | 68.76 |
| 3 - Associate professional and technical occupations | 261 | 142 | 54.23 | 101 | 52 | 53.10 | 160 | 90 | 54.82 |
| 4 - Administrative and secretarial occupations | 133 | 69 | 55.53 | 105 | 55 | 54.32 | 28 | 14 | 59.32 |
| 5 - Skilled trades occupations | 136 | 88 | 61.97 | 12 | 7 | 50.53 | 124 | 81 | 63.18 |
| 6 - Caring, leisure and other service occupations | 138 | 91 | 70.76 | 119 | 83 | 74.09 | 19 | 8 | 46.33 |
| 7 - Sales and customer service occupations | 104 | 55 | 50.83 | 73 | 42 | 56.59 | 31 | 13 | 38.79 |
| 8 - Process, plant and machine operatives | 109 | 72 | 68.30 | 14 | 6 | 46.19 | 95 | 66 | 71.97 |
| 9 - Elementary occupations | 127 | 71 | 60.48 | 68 | 38 | 65.22 | 59 | 33 | 54.87 |
|  |  |  |  |  |  |  |  |  |  |
| Overall | 1467 | 883 |  | 706 | 424 |  | 761 | 459 |  |
|  |  |  |  |  |  |  |  |  |  |

^a^ The population includes all those who were present and employed at both time periods, and who were aged 40-64 years at wave 10, excluding those interviewed in 2020.

^b^ Weighted percentages are reported.

Source: UKLHS wave 3 (2011-2013) and wave 10 (2018-2020).

# **Table S3** Covariates included in the statistical analysis

| Variable | Coding/category description | Data source ^a^ |
| --- | --- | --- |
| *Socio-demographic variables:* | | |
| Age (years) | Restricted cubic spline with three knots (age 42, 53 and 62 years) | CHI register |
| Sex | Women, Men | CHI register |
| Ethnicity | Non-White, White | NRS 2011 Census |
|  | | |
| *Household-level variables:* | | |
| Housing tenure | Owned outright, Owned with mortgage,  Social rented, Private rented, Owned/not known | NRS 2011 Census |
| Household size | 1-2 people, 3-4 people, 5-6 people, 7+ people | OS UPRN |
| Household with children | No children, At least one child 0-11 years, At least one child 12-17 years | CHI register and  OS UPRN |
| Whether multigenerational household | Binary (Yes, No) variable identifying household with at least one person aged 65 or more years and a person at least 20 years younger | CHI register and  OS UPRN |
|  | | |
| *Occupational exposure measures:* | | |
| Exposure to disease | Standardised score ranging from 0 (lowest frequency of exposure to disease or infection) to 1 (maximum frequency of exposure to disease or infection) | US DOL ETA O*NET survey data |
| Proximity to others | Standardised score ranging from 0 (no physical proximity to others) to 1 (maximum physical proximity to others) | US DOL ETA O*NET survey data |
| Environmentally controlled indoor conditions | Standardised score ranging from 0 (lowest frequency of working in environmentally controlled indoor conditions) to 1 (maximum frequency of working in environmentally controlled indoor conditions) | US DOL ETA O*NET survey data |
|  | | |
| *Health-related conditions:* | | |
| Learning disability or difficulty | Categorical variable (Yes, No, Not known) capturing whether a person has learning disability (e.g. Down’s syndrome) or learning difficulty (e.g. dyslexia) | NRS 2011 Census |
| Whether shielding | Yes, No | PHS Shielding Patient List |
| Pre-pandemic health conditions | Binary (Yes, No) variables identifying whether a person has: cancer and immunosuppression, cardiovascular conditions, diabetes, hypertension, respiratory conditions, and other conditions | GP cluster data from PHS COVID-19 Research Database |
|  |  |  |

^a^ Abbreviations: CHI: Community Health Index; NRS: National Records of Scotland; OS UPRN: Ordnance Survey Unique Property Reference Number; US DOL ETA: US Department of Labor, Employment and Training Administration; O*NET: Occupational Information Network; PHS: Public Health Scotland; GP: General Practitioner.

# **Table S4** Description of the groups of people and conditions included in the shielding patient list

| No. | Group |
| --- | --- |
|  |  |
| 1. | Recipients of solid organ transplant |
| 2. | People receiving chemotherapy or antibody treatment for cancer, including immunotherapy |
| 3. | People receiving radical radiotherapy for lung cancer |
| 4. | People receiving cancer treatments affecting immune system (protein kinase inhibitors or PARP inhibitors) |
| 5. | People with blood or bone marrow cancer (leukemia, lymphoma or myeloma) |
| 6. | Recipients of bone marrow or stem cell transplant in past 6 months or taking immunosuppressant medicine |
| 7. | People with severe lung condition (cystic fibrosis, severe asthma or COPD) |
| 8. | People with high risk of getting infections (SCID or sickle cell) |
| 9. | People taking medicine that makes them at higher risk of infections (steroids or immunosuppressant medicine) |
| 10. | People with serious heart condition or pregnant |
| 11. | People with problem with their spleen or spleen has been removed (splenectomy) |
| 12. | People with Down's syndrome |
| 13. | Recipient of dialysis or severe long-term kindney disease (stage 5) |
|  |  |

Source: Public Health Scotland. Search criteria for highest risk patients for inclusion to the shielding list. 2020. <https://hpspubsrep.blob.core.windows.net/hps-website/nss/3008/documents/1_covid-19-search-criteria-highest-risk-patients.pdf>. Accessed 15 August 2023.

**Table S5** Clusters and Read codes used to derive pre-pandemic health conditions from primary care records ^a^

| Pre-pandemic health condition | Cluster code | Cluster description | Read code ^b^ |
| --- | --- | --- | --- |
| Cancer and immunosuppression | EAVE_HAEMAT_MALIGNANCY | Haematological malignancies | 'B6%' |
|  | EAVE_OTHER_MALIGNANCY | Other suspected malignancy | '1J0%' (not 1J0F.) |
|  | EAVE_IMMUNOSUPPRESSION | Immunosuppression | 'PK01.', 'PK06.', 'G74y6', '14N7.', '7840%', 'D4154', 'D4156', '2J30.', '2J31.', 'A788%', A789%', '43C3.', 'AyuC%', 'C332.', 'C332z', 'C333%', 'D41y1' |
|  | EAVE_TRANSPLANT | Transplantation | '7450%', '7800%', '7830%', '7900%', '7901%', '8C31.' |
|  |  |  |  |
| Cardiovascular conditions | EAVE_CHRONIC_HEART_DIS | Chronic heart disease | '33BA.', 'G1%', 'G21%', 'G220.', 'G222.', 'G23%', 'G3%', 'G41%', 'G54%', 'G55%', 'G58%', 'G5y1.', 'G5y3%', 'G5y4%', 'G5y6.', 'G5y7.', 'G5y8.', 'G5yy2', 'G5yy6', 'G5yy9', 'G5yyA', 'Gyu1%', 'Gyu3%', 'Gyu4%', 'Gyu55', 'Gyu56', 'Gyu57', 'Gyu58', 'Gyu59', 'Gyu5A', 'Gyu5B', 'Gyu5C', 'Gyu5D', 'Gyu5M', 'Gyu5N', 'Gyu5P', 'Gyu5Q', 'Gyu5R', 'Gyu5S', 'Gyu5T', 'P5%', 'P60%', 'P61%', 'P62%', 'P63%', 'P64%', 'P65%', 'P66%', 'P67%', 'P68%', 'P6W%', 'P6X%', 'P6y..', 'P6y0%', 'P6y1%', 'P6y2%', 'P6y3%', 'P6y63', 'P6y64', 'P6y6z', 'P6yy%', 'P6z..', 'P6z2.', 'P6z3.' |
|  | EAVE_STROKE_TIA | Stroke/Transient Ischaemic Attack (TIA) | 'G61..', 'G610%', 'G611%', 'G612%', 'G613%', 'G614%', 'G615%', 'G616%', 'G618.', 'G61X.', 'G61X0', 'G61X1', 'G61z.', 'G63y0', 'G63y1', 'G64%', 'G66%', 'G6760.', 'G6W..', 'G6X..', 'Gyu62', 'Gyu63', 'Gyu64', 'Gyu65', 'Gyu66', 'Gyu6C', 'Gyu6F', 'Gyu6G', 'G65..', 'G650%', 'G651%', 'G6510', 'G652%', 'G653%', 'G654%', 'G656.', 'G65y.', 'G65z%', 'G65z0', 'G65z1', 'G65zz', 'F4236', 'Fyu55' |
|  | EAVE_PER_VASCULAR_DIS | Peripheral vascular disease | 'G73%', 'Gyu74' |
|  |  |  |  |
| Diabetes ^c^ | EAVE_DIABETES | Diabetes | 'C10%.', 'Cyu2%', 'L1805', 'L1806', 'L1807', 'L180X', 'Lyu29.' |
|  |  |  |  |
| Hypertension ^d^ | EAVE_HYPERTENSION | Hypertension | 'G2%' |
|  |  |  |  |
| Respiratory conditions | EAVE_CHRONIC_RESP_DIS | Chronic respiratory disease (including asthma and Chronic Obstructive Pulmonary Disease (COPD)) | 'A115.', 'H3%', 'C370.', 'H40%', 'H41%', 'H42%', 'H43%', 'H44%', 'H45%', 'H46..', 'H460.', 'H460z', 'H464%', 'H46z% H47y0', 'H48% H4y%', 'H4z%', 'H5410.', 'H55%', 'H563%', 'H57%', 'H582.', 'H583.', 'H591.', 'H592.', 'H593.', 'Hyu3%', 'Hyu40', 'Hyu41', 'Hyu48', 'Hyu5%', 'Q3170' |
|  |  |  |  |
| Other conditions | EAVE_CHRONIC_LIVER_DIS | Chronic liver disease | 'A707%', 'J6...', 'J61%', 'J62y.', 'J62z.', 'J6353', 'J6354', 'J6355', 'J6356', 'J63B.', 'PB61%', 'PB63%', 'PB6y1', 'J623.', 'J624.', 'J625.', 'J63A.', 'J6617', 'Jyu71', 'SP143' |
|  |  |  |  |
|  | EAVE_CHRONIC_PANCREATITIS | Chronic pancreatitis | 'J641%', 'Jyu84' |
|  |  |  |  |
|  | EAVE_DEMENTIA | Dementia | 'E00%', 'E012.', 'E02y1', 'E041.', 'Eu00%', 'Eu01%', 'Eu02%', 'Eu03%', 'Eu04%' |
|  |  |  |  |
|  | EAVE_DEPRESSION | Depression | 'E11%', 'E130.', 'E135.', 'E2003', 'E204.', 'E2112', 'E290%', 'E291%', 'E2B%' |
|  |  |  |  |
|  | EAVE_MS_DEGEN_DIS | Multiple sclerosis (MS) and degenerative disease | 'F20%', 'F21%', 'F22%', 'F23%', 'F24..', 'F240%', 'F241%', 'F242.', 'F24y%', 'F24z.', 'F2A..', 'F2Az.', 'Fyu9%', 'G669.', 'F1...', 'F10%', 'F11%', 'F12%', 'F13..', 'F130%', 'F1322', 'F134.', 'F135.', 'F1350', 'F135z', 'F136%', 'F137.', 'F1370', 'F1371', 'F137y', 'F137z', 'F13A.', 'F13X.', 'F14%', 'F15%', 'F16..', 'F160%', 'F161%', 'F162.', 'F163.', 'F1631', 'F163z', 'F16y%', 'F16z.', 'F17..', 'F174.', 'F1y..', 'F1z..' |
|  |  |  |  |
|  | EAVE_MYONEURAL_DIS | Myoneural disorders | 'D41y1', 'D41y2', 'F38%', 'Fyu8.', 'Fyu80', 'Fyu83', 'Fyu84', 'Fyu85' |
|  |  |  |  |
|  | EAVE_RHEUMATOLOGICAL_DIS | Rheumatological disorders | 'N.%' |
|  |  |  |  |

^a^ Primary care records covering 940 general practices across Scotland were provided by Albsoft ltd through Public Health Scotland’s COVID-19 Research Database; Cluster codes were developed by the Early Pandemic Evaluation and Enhanced Surveillance of COVID-19 (EAVE II) research team; Additional information can be found at <https://github.com/EAVE-II/EAVE-II-data-dictionary>.

^b^ Read codes Version 2 (Scottish edition) are the national coding system in Scottish general practice and primary care for recording clinical information arising from a patient encounter; more information can be found at [https://isd.digital.nhs.uk/trud/user/guest/group/0/home.](https://isd.digital.nhs.uk/trud/user/guest/group/0/home)

^c^ This condition does not include patients for whom diabetes was resolved (Read code '21263').

^d^ This condition does not include patients for whom hypertension was resolved (Read code '21261').

# **Figure S1** Directed Acyclic Graph (DAG) showing the hypothesised relationships between occupation, the outcome variables and other covariates included in the statistical analysis


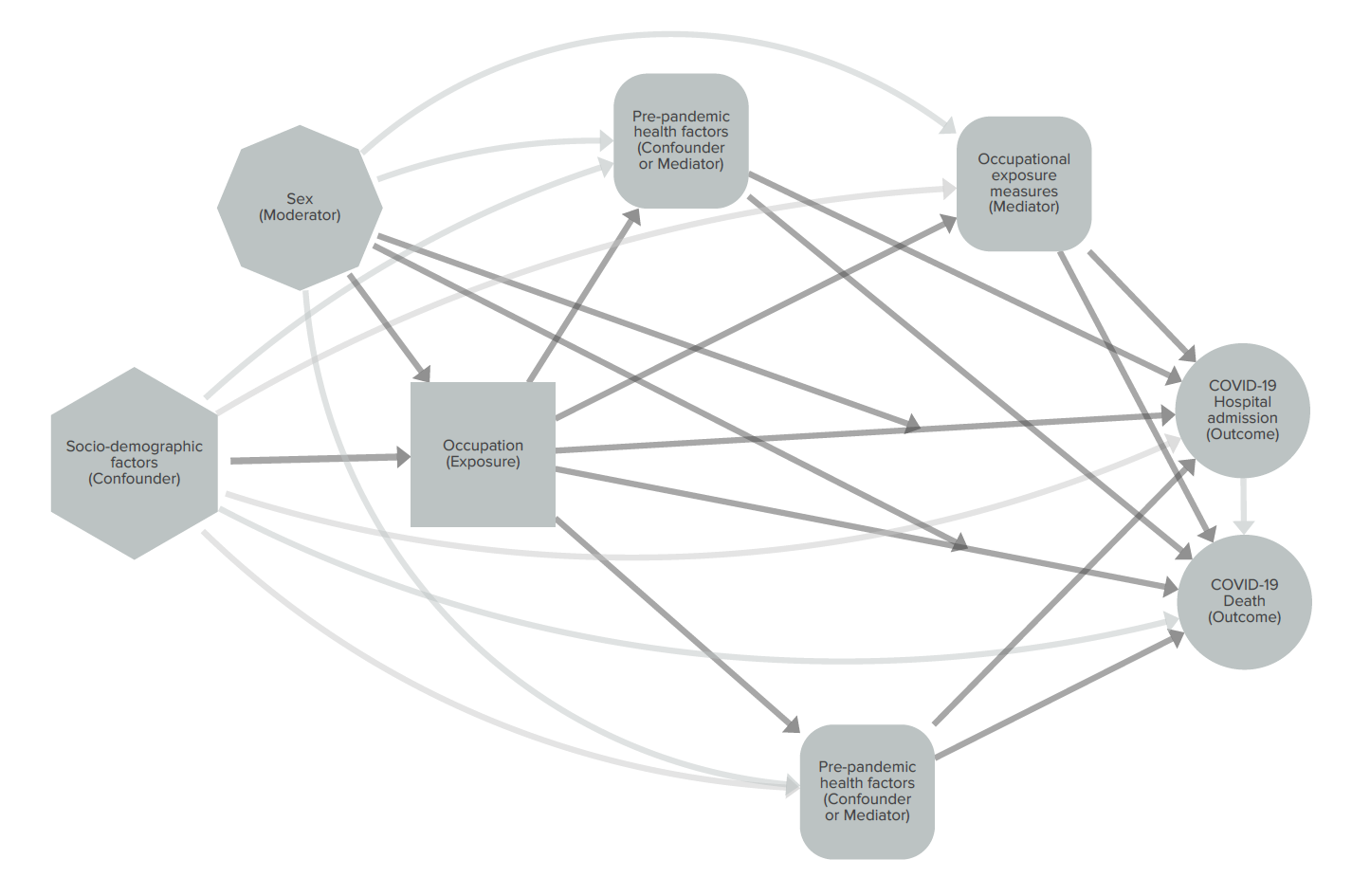


Note: Shapes indicate the different roles (in parentheses) played by each variable in the causal diagram; Socio-demographic factors (Confounder): age and ethnicity; Household-level factors (Confounder or Mediator): housing tenure, household size, whether children in the household, whether multigenerational household; Pre-pandemic health factors (Confounder or Mediator): learning disability or difficulty, whether shielding, pre-pandemic health conditions; Occupational exposure measures (Mediator): exposure to disease, proximity to others, and environmentally controlled indoor conditions; Occupation (Exposure): SOC 2010 codes; COVID-19 Hospital admission (Outcome); COVID-19 Death (Outcome). The main directed arrows between variables are highlighted in a darker shade.

**Table S6** Full Cox proportional hazards models of risk of COVID-19 hospital admission for women and men aged 40-64 years in Scotland ^a^

|  | | Model 1 | |  | Model 2 | |  | Model 3 | |  | Model 4 | |
| --- | --- | --- | --- | --- | --- | --- | --- | --- | --- | --- | --- | --- |
|  | | Women | Men |  | Women | Men |  | Women | Men |  | Women | Men |
| Covariate | | HR (95%CI) ^b^ | HR (95%CI) ^b^ |  | HR (95%CI) ^b^ | HR (95%CI) ^b^ |  | HR (95%CI) ^b^ | HR (95%CI) ^b^ |  | HR (95%CI) ^b^ | HR (95%CI) ^b^ |
| *Age (years; restricted cubic spline):* | | | | | | | | | | | | |
| Age parameter 1 | | **1.07 (1.05-1.09)** | **1.09 (1.07-1.10)** |  | **1.07 (1.05-1.09)** | **1.09 (1.07-1.10)** |  | **1.06 (1.04-1.07)** | **1.07 (1.05-1.09)** |  | **1.06 (1.04-1.07)** | **1.07 (1.05-1.09)** |
| Age parameter 2 | | **0.97 (0.95-0.99)** | **0.98 (0.96-0.99)** |  | **0.97 (0.96-0.99)** | **0.98 (0.96-0.99)** |  | **0.97 (0.95-0.98)** | **0.97 (0.96-0.99)** |  | **0.97 (0.95-0.98)** | **0.97 (0.96-0.99)** |
| *Ethnicity (reference category: White):* | | | | | | | | | | | | |
| Not white | | 1.09 (0.90-1.30) | **1.28 (1.06-1.52)** |  | **1.72 (1.43-2.06)** | **1.91 (1.61-2.25)** |  | **1.88 (1.57-2.25)** | **2.11 (1.80-2.47)** |  | **1.88 (1.57-2.25)** | **2.15 (1.84-2.52)** |
| *Occupation (SOC 2010 codes; reference category: 1 – Managers, directors and senior officials):* | | | | | | | | | | | | |
| 2 - | Professional occupations | **0.58 (0.44-0.76)** | **0.49 (0.39-0.61)** |  | **0.60 (0.46-0.79)** | **0.50 (0.40-0.62)** |  | **0.63 (0.48-0.83)** | **0.53 (0.42-0.66)** |  | **0.55 (0.41-0.72)** | **0.52 (0.42-0.65)** |
| 22 - | Health professionals | **1.63 (1.33-2.01)** | 1.25 (0.95-1.63) |  | **1.64 (1.33-2.02)** | 1.21 (0.93-1.58) |  | **1.65 (1.34-2.03)** | 1.23 (0.94-1.61) |  | 0.92 (0.68-1.24) | 0.87 (0.64-1.18) |
| 24 - | Business, media and public service professionals | **0.49 (0.34-0.71)** | **0.61 (0.48-0.78)** |  | **0.51 (0.35-0.74)** | **0.63 (0.49-0.80)** |  | **0.53 (0.36-0.76)** | **0.67 (0.52-0.85)** |  | **0.52 (0.36-0.75)** | **0.66 (0.52-0.85)** |
| 3 - | Associate professional and technical occupations | 0.83 (0.63-1.10) | 0.86 (0.69-1.06) |  | 0.82 (0.62-1.09) | 0.87 (0.70-1.07) |  | 0.82 (0.62-1.09) | 0.89 (0.72-1.10) |  | **0.63 (0.46-0.85)** | 0.84 (0.67-1.04) |
|  |  |  |  |  |  |  |  |  |  |  |  |  |
| 33 - | Protective service occupations | - | 1.24 (0.99-1.56) |  | - | 1.25 (0.99-1.58) |  | - | **1.31 (1.04-1.64)** |  | - | 1.12 (0.87-1.45) |
| 35 - | Business and public service associate professionals | **0.60 (0.45-0.81)** | **0.66 (0.53-0.82)** |  | **0.62 (0.46-0.83)** | **0.67 (0.54-0.84)** |  | **0.63 (0.47-0.85)** | **0.69 (0.55-0.86)** |  | **0.65 (0.48-0.87)** | **0.69 (0.55-0.85)** |
|  |  |  |  |  |  |  |  |  |  |  |  |  |
| 41 - | Administrative occupations | 1.06 (0.87-1.30) | 0.97 (0.78-1.22) |  | 1.06 (0.86-1.30) | 0.98 (0.78-1.23) |  | 1.05 (0.86-1.29) | 0.97 (0.78-1.22) |  | 1.00 (0.81-1.23) | 0.94 (0.75-1.18) |
| 42 - | Secretarial and related occupations | 1.13 (0.88-1.45) | - |  | 1.13 (0.88-1.45) | - |  | 1.15 (0.90-1.48) | - |  | 1.12 (0.88-1.44) | - |
| 5 - | Skilled trades occupations | 0.85 (0.52-1.41) | 0.87 (0.74-1.03) |  | 0.84 (0.51-1.39) | 0.88 (0.75-1.04) |  | 0.80 (0.48-1.33) | 0.90 (0.77-1.06) |  | 0.75 (0.45-1.28) | 0.92 (0.75-1.12) |
| 53 - | Skilled construction and building trades | - | 0.99 (0.84-1.18) |  | - | 0.97 (0.82-1.15) |  | - | 1.00 (0.84-1.19) |  | - | 1.01 (0.80-1.27) |
| 543 - | Food preparation and hospitality trades | **1.41 (1.01-1.96)** | 1.17 (0.90-1.52) |  | 1.24 (0.89-1.73) | 1.00 (0.77-1.31) |  | 1.14 (0.81-1.59) | 0.91 (0.69-1.18) |  | 0.99 (0.69-1.42) | 0.88 (0.66-1.17) |
| 6 - | Caring, leisure and other service occupations | **1.25 (1.01-1.56)** | 1.21 (0.90-1.64) |  | 1.20 (0.96-1.50) | 1.15 (0.85-1.57) |  | 1.19 (0.96-1.49) | 1.11 (0.82-1.51) |  | 0.90 (0.68-1.19) | 1.01 (0.74-1.38) |
| 614 - | Caring personal services | **2.26 (1.79-2.87)** | **2.45 (1.80-3.34)** |  | **2.08 (1.64-2.64)** | **2.33 (1.71-3.18)** |  | **1.96 (1.54-2.48)** | **2.15 (1.58-2.93)** |  | 1.07 (0.76-1.51) | **1.57 (1.11-2.22)** |
| 6145 - | Care workers and home workers | **2.16 (1.75-2.66)** | **1.97 (1.48-2.62)** |  | **1.88 (1.52-2.32)** | **1.78 (1.34-2.37)** |  | **1.69 (1.37-2.08)** | **1.63 (1.22-2.16)** |  | 1.12 (0.80-1.56) | **1.38 (1.00-1.91)** |
| 71 - | Sales occupations | **1.43 (1.16-1.76)** | 1.27 (0.99-1.62) |  | **1.26 (1.02-1.56)** | 1.16 (0.90-1.48) |  | 1.18 (0.96-1.46) | 1.07 (0.84-1.37) |  | 1.06 (0.84-1.34) | 1.06 (0.83-1.37) |
| 72 - | Customer service occupations | **1.93 (1.47-2.54)** | 1.26 (0.88-1.79) |  | **1.82 (1.39-2.39)** | 1.22 (0.86-1.74) |  | **1.70 (1.29-2.23)** | 1.14 (0.80-1.62) |  | **1.61 (1.22-2.11)** | 1.09 (0.77-1.56) |
| 81 - | Process, plant and machine operatives | **2.20 (1.61-3.00)** | 1.05 (0.85-1.29) |  | **1.84 (1.35-2.52)** | 1.01 (0.82-1.25) |  | **1.57 (1.14-2.14)** | 1.00 (0.81-1.24) |  | **1.49 (1.05-2.11)** | 1.02 (0.80-1.29) |
| 811- | Process plant operatives | **1.72 (1.18-2.51)** | 1.13 (0.84-1.52) |  | 1.45 (1.00-2.12) | 1.07 (0.80-1.44) |  | 1.31 (0.90-1.91) | 1.09 (0.81-1.46) |  | 1.24 (0.82-1.88) | 1.11 (0.82-1.51) |
| 82 - | Transport and mobile machine drivers and operatives | 1.43 (0.75-2.72) | **1.34 (1.12-1.60)** |  | 1.31 (0.69-2.49) | **1.26 (1.05-1.51)** |  | 1.18 (0.62-2.25) | **1.20 (1.00-1.44)** |  | 0.92 (0.46-1.83) | 1.16 (0.93-1.45) |
|  |  |  |  |  |  |  |  |  |  |  |  |  |
| 8211 - | Large goods vehicle drivers | - | **1.29 (1.01-1.63)** |  | - | 1.24 (0.98-1.58) |  | - | 1.23 (0.97-1.57) |  | - | 1.24 (0.96-1.61) |
| 8214 - | Taxi and cab drivers and chauffeurs | - | **2.37 (1.91-2.95)** |  | - | **2.16 (1.73-2.69)** |  | - | **1.92 (1.54-2.39)** |  | - | **1.84 (1.42-2.40)** |
| 9 - | Elementary occupations | 1.21 (0.87-1.69) | **1.20 (1.00-1.43)** |  | 1.06 (0.76-1.48) | 1.10 (0.92-1.32) |  | 0.99 (0.71-1.38) | 1.07 (0.89-1.28) |  | 0.88 (0.62-1.26) | 1.03 (0.85-1.25) |
| 91 - | Elementary trades and related occupations | **1.62 (1.10-2.38)** | 1.03 (0.82-1.29) |  | 1.34 (0.91-1.97) | 0.94 (0.75-1.19) |  | 1.19 (0.81-1.75) | 0.92 (0.73-1.16) |  | 1.08 (0.69-1.69) | 0.94 (0.72-1.24) |
| 927 - | Other elementary services occupations | **1.56 (1.22-2.00)** | **1.59 (1.18-2.15)** |  | **1.31 (1.02-1.68)** | **1.39 (1.02-1.88)** |  | 1.20 (0.93-1.54) | 1.25 (0.92-1.69) |  | 1.02 (0.76-1.37) | 1.20 (0.88-1.64) |
| 9233 | Cleaners and domestics | **1.90 (1.52-2.37)** | 0.90 (0.57-1.44) |  | **1.53 (1.21-1.91)** | 0.78 (0.49-1.24) |  | **1.35 (1.08-1.70)** | 0.74 (0.46-1.18) |  | 1.16 (0.88-1.53) | 0.68 (0.42-1.08) |
| No code required | | **1.82 (1.43-2.32)** | 0.94 (0.76-1.16) |  | **1.98 (1.55-2.52)** | **1.96 (1.55-2.47)** |  | **1.53 (1.20-1.95)** | **1.90 (1.52-2.38)** |  | 1.77 (0.98-3.22) | **2.20 (1.54-3.13)** |

| *Housing tenure (reference category: Owned outright):* | | | | | | | | | | | |
| --- | --- | --- | --- | --- | --- | --- | --- | --- | --- | --- | --- |
| Owned with mortgage |  |  |  | **1.42 (1.25-1.62)** | **1.48 (1.31-1.67)** |  | **1.35 (1.18-1.53)** | **1.45 (1.28-1.64)** |  | **1.34 (1.18-1.53)** | **1.43 (1.27-1.62)** |
| Social rented |  |  |  | **2.15 (1.86-2.47)** | **1.96 (1.71-2.25)** |  | **1.57 (1.36-1.82)** | **1.55 (1.35-1.78)** |  | **1.58 (1.37-1.83)** | **1.57 (1.37-1.81)** |
| Private rented |  |  |  | **1.29 (1.07-1.57)** | **1.22 (1.02-1.45)** |  | 1.12 (0.92-1.35) | 1.10 (0.92-1.31) |  | 1.12 (0.93-1.36) | 1.11 (0.93-1.33) |
| Other/not known |  |  |  | **0.62 (0.47-0.81)** | **0.34 (0.26-0.46)** |  | **2.45 (1.45-4.12)** | **1.93 (1.35-2.77)** |  | **2.39 (1.41-4.02)** | **1.91 (1.33-2.74)** |
| *Household size (UPRN; reference category: 1-2 people):* | | | | | | | | | | | |
| 3-4 people |  |  |  | 0.99 (0.90-1.08) | **1.15 (1.06-1.25)** |  | 1.03 (0.94-1.12) | **1.20 (1.10-1.30)** |  | 1.01 (0.92-1.10) | **1.14 (1.05-1.24)** |
| 5-6 people |  |  |  | 1.10 (0.95-1.28) | **1.31 (1.15-1.50)** |  | 1.15 (0.99-1.33) | **1.35 (1.18-1.54)** |  | 1.11 (0.96-1.29) | **1.27 (1.11-1.46)** |
| 7+ people |  |  |  | **1.67 (1.33-2.09)** | **2.25 (1.88-2.70)** |  | **1.64 (1.31-2.06)** | **2.12 (1.76-2.55)** |  | **1.51 (1.19-1.90)** | **1.92 (1.59-2.31)** |
| *Household with children (UPRN; reference category: No children):* | | | | | | | | | | | |
| At least one child (0-11 years) |  |  |  | 0.90 (0.77-1.05) | **0.81 (0.70-0.93)** |  | 0.89 (0.77-1.04) | **0.82 (0.71-0.95)** |  | 0.90 (0.77-1.05) | **0.83 (0.72-0.96)** |
| At least one child (12-17 years) |  |  |  | 0.92 (0.81-1.05) | **0.83 (0.73-0.95)** |  | 0.93 (0.81-1.06) | **0.85 (0.75-0.97)** |  | 0.94 (0.82-1.07) | **0.85 (0.75-0.97)** |
| *Whether multigenerational household (UPRN; reference category: No):* | | | | | | | | | | | |
| Yes |  |  |  | 1.06 (0.93-1.22) | 1.09 (0.96-1.24) |  | 1.03 (0.89-1.18) | 1.06 (0.93-1.21) |  | 0.99 (0.86-1.14) | 1.02 (0.90-1.17) |
| *Learning disability or difficulty (reference category: No):* | | | | | | | | | | | |
| Yes |  |  |  |  |  |  | **1.45 (1.15-1.81)** | **1.41 (1.16-1.71)** |  | **1.44 (1.15-1.81)** | **1.42 (1.17-1.72)** |
| Not known |  |  |  |  |  |  | **0.25 (0.14-0.44)** | **0.15 (0.10-0.22)** |  | **0.25 (0.14-0.43)** | **0.14 (0.09-0.21)** |
| *Whether shielding (reference category: No):* | | | | | | | | | | | |
| Yes |  |  |  |  |  |  | **3.24 (2.85-3.67)** | **3.25(2.82-3.74)** |  | **3.24 (2.86-3.68)** | **3.26 (2.83-3.76)** |
| *Pre-pandemic health conditions (reference category for each covariate: No):* | | | | | | | | | | | |
| Cancer and immunosuppression |  |  |  |  |  |  | **1.67 (1.36-2.05)** | **1.46 (1.19-1.81)** |  | **1.66 (1.36-2.04)** | **1.46 (1.18-1.80)** |
| Cardiovascular conditions |  |  |  |  |  |  | **1.53 (1.36-1.71)** | **1.58 (1.43-1.73)** |  | **1.53 (1.36-1.71)** | **1.58 (1.43-1.73)** |
| Diabetes |  |  |  |  |  |  | **2.26 (2.03-2.51)** | **2.16 (1.97-2.37)** |  | **2.25 (2.02-2.51)** | **2.16 (1.97-2.37)** |
| Hypertension |  |  |  |  |  |  | **1.44 (1.31-1.58)** | **1.37 (1.26-1.49)** |  | **1.44 (1.31-1.58)** | **1.37 (1.26-1.49)** |
| Respiratory conditions |  |  |  |  |  |  | **1.40 (1.28-1.54)** | **1.16 (1.05-1.28)** |  | **1.40 (1.28-1.54)** | **1.16 (1.05-1.28)** |
| Other conditions |  |  |  |  |  |  | **1.36 (1.25-1.47)** | **1.38 (1.26-1.50)** |  | **1.36 (1.25-1.47)** | **1.38 (1.26-1.50)** |
| *Occupational exposure measures (O*NET scores 0-1):* | | | | | | | | | | | |
| Exposure to disease |  |  |  |  |  |  |  |  |  | **1.81 (1.42-2.31)** | **1.67 (1.43-1.95)** |
| Proximity to others |  |  |  |  |  |  |  |  |  | **1.71 (1.03-2.87)** | 1.06 (0.74-1.52) |
| Environmentally controlled indoor conditions |  |  |  |  |  |  |  |  |  | 0.81 (0.44-1.50) | 1.09 (0.82-1.44) |
|  |  |  |  |  |  |  |  |  |  |  |  |
| COVID-19 hospital admissions (n) | 2,820 | 3,140 |  | 2,820 | 3,140 |  | 2,820 | 3,140 |  | 2,820 | 3,140 |
| Total persons (n) | 859,800 | 840,870 |  | 859,800 | 840,870 |  | 859,800 | 840,870 |  | 859,800 | 840,870 |
| Total episodes (n) | 937,810 | 914,600 |  | 937,810 | 914,600 |  | 937,810 | 914,600 |  | 937,810 | 914,600 |
| AIC | 76,175 | 84,755 |  | 75,982 | 84,468 |  | 74,895 | 83,428 |  | 74,871 | 83,392 |
| BIC | 76,504 | 85,095 |  | 76,429 | 85,925 |  | 75,447 | 83,991 |  | 75,471 | 83,990 |
| Likelihood Ratio (LR) test p-value | <0.001 | <0.001 |  | <0.001 | <0.001 |  | <0.001 | <0.001 |  | <0.001 | <0.001 |

^a^ Hospital admissions occurring between 1 March 2020 and 31 January 2021.

^b^ Hazard Ratios (HR) and 95% Confidence Intervals (CI) are reported; Estimates are not reported if there were less than 10 hospital admissions; HRs greater than 1 with p < 0.05 are highlighted in bold, while HRs less than 1 with p < 0.05 are shown in white within shaded grey cells.

**Table S7** Full Cox proportional hazards models of risk of COVID-19 related death for women and men aged 40-64 years in Scotland ^a^

|  | | Model 1 | |  | Model 2 | |  | Model 3 | |  | Model 4 | |
| --- | --- | --- | --- | --- | --- | --- | --- | --- | --- | --- | --- | --- |
|  | | Women | Men |  | Women | Men |  | Women | Men |  | Women | Men |
| Covariate | | HR (95%CI) ^b^ | HR (95%CI) ^b^ |  | HR (95%CI) ^b^ | HR (95%CI) ^b^ |  | HR (95%CI) ^b^ | HR (95%CI) ^b^ |  | HR (95%CI) ^b^ | HR (95%CI) ^b^ |
| *Age (years; restricted cubic spline):* | | | | | | | | | | | | |
| Age parameter 1 | | **1.08 (1.02-1.14)** | **1.13 (1.07-1.19)** |  | **1.08 (1.02-1.15)** | **1.13 (1.07-1.20)** |  | 1.06 (1.00-1.12) | **1.11 (1.05-1.18)** |  | 1.06 (0.99-1.12) | **1.11 (1.05-1.17)** |
| Age parameter 2 | | 1.02 (0.96-1.08) | 1.01 (0.96-1.06) |  | 1.01 (0.96-1.07) | 1.01 (0.96-1.06) |  | 1.01 (0.95-1.06) | 1.00 (0.95-1.05) |  | 1.01 (0.95-1.06) | 1.00 (0.95-1.05) |
| *Ethnicity (reference category: White):* | | | | | | | | | | | | |
| Not white | | **0.43 (0.24-0.75)** | **0.34 (0.22-0.52)** |  | 1.21 (0.66-2.23) | 0.71 (0.41-1.23) |  | 1.52 (0.85-2.73) | **1.81 (1.15-2.87)** |  | 1.57 (0.88-2.80) | **1.87 (1.18-2.96)** |
| *Occupation (SOC 2010 codes; reference category: 1 – Managers, directors and senior officials):* | | | | | | | | | | | | |
| 2 - | Professional occupations | 0.52 (0.23-1.17) | 0.53 (0.27-1.01) |  | 0.57 (0.25-1.29) | 0.54 (0.28-1.05) |  | 0.62 (0.27-1.41) | 0.60 (0.31-1.15) |  | 0.49 (0.21-1.15) | 0.59 (0.30-1.13) |
| 22 - | Health professionals | 0.63 (0.29-1.35) | 0.19 (0.03-1.37) |  | 0.66 (0.31-1.43) | 0.19 (0.03-1.35) |  | 0.67 (0.31-1.44) | 0.19 (0.03-1.36) |  | **0.28 (0.10-0.78)** | 0.15 (0.02-1.11) |
| 24 - | Business, media and public service professionals | - | 0.68 (0.34-1.38) |  | - | 0.71 (0.35-1.44) |  | - | 0.79 (0.39-1.59) |  | - | 0.78 (0.38-1.57) |
| 3 - | Associate professional and technical occupations | 0.37 (0.13-1.12) | 1.00 (0.61-1.98) |  | 0.37 (0.12-1.11) | 1.05 (0.58-1.90) |  | 0.37 (0.12-1.12) | 1.10 (0.61-2.00) |  | **0.24 (0.07-0.78)** | 1.10 (0.60-2.01) |
|  |  |  |  |  |  |  |  |  |  |  |  |  |
| 33 - | Protective service occupations | - | 1.42 (0.72-2.79) |  | - | 1.38 (0.70-2.72) |  | - | 1.49 (0.76-2.94) |  | - | 1.79 (0.85-3.78) |
| 35 - | Business and public service associate professionals | **0.27 (0.08-0.92)** | 0.89 (0.49-1.61) |  | **0.29 (0.08-0.99)** | 0.90 (0.50-1.63) |  | 0.30 (0.09-1.04) | 0.95 (0.53-1.72) |  | 0.31 (0.09-1.07) | 0.96 (0.53-1.74) |
|  |  |  |  |  |  |  |  |  |  |  |  |  |
| 41 - | Administrative occupations | 1.00 (0.55-1.82) | 1.54 (0.87-2.72) |  | 1.02 (0.56-1.85) | 1.43 (0.81-2.53) |  | 1.03 (0.57-1.87) | 1.45 (0.82-2.56) |  | 0.94 (0.51-1.73) | 1.48 (0.83-2.64) |
| 42 - | Secretarial and related occupations | 0.98 (0.46-2.07) | - |  | 0.99 (0.47-2.09) | - |  | 1.04 (0.49-2.20) | - |  | 1.00 (0.47-2.12) | - |
| 5 - | Skilled trades occupations | 0.82 (0.19-3.55) | 0.83 (0.51-1.35) |  | 0.74 (0.17-3.23) | 0.76 (0.46-1.24) |  | 0.67 (0.15-2.90) | 0.79 (0.49-1.30) |  | 0.59 (0.13-2.75) | 1.12 (0.63-1.97) |
| 53 - | Skilled construction and building trades | - | 1.29 (0.80-2.08) |  | - | 1.13 (0.70-1.83) |  | - | 1.18 (0.73-1.92) |  | - | 1.80 (0.97-3.34) |
| 543 - | Food preparation and hospitality trades | 1.02 (0.34-3.07) | **2.12 (1.08-4.18)** |  | 0.82 (0.27-2.45) | 1.53 (0.77-3.04) |  | 0.71 (0.24-2.12) | 1.21 (0.61-2.41) |  | 0.57 (0.18-1.82) | 1.31 (0.63-2.73) |
| 6 - | Caring, leisure and other service occupations | 0.59 (0.27-1.29) | 1.30 (0.55-3.08) |  | 0.55 (0.25-1.22) | 1.05 (0.44-2.50) |  | 0.55 (0.25-1.22) | 0.99 (0.42-2.37) |  | **0.36 (0.14-0.92)** | 1.02 (0.42-2.47) |
| 614 - | Caring personal services | 1.32 (0.60-2.90) | 0.92 (0.22-3.83) |  | 1.18 (0.53-2.61) | 0.79 (0.19-3.29) |  | 1.09 (0.49-2.41) | 0.72 (0.17-2.99) |  | 0.44 (0.14-1.32) | 0.66 (0.15-2.92) |
| 6145 - | Care workers and home workers | **2.00 (1.08-3.69)** | 1.61 (0.63-4.12) |  | 1.61 (0.87-3.00) | 1.23 (0.48-3.16) |  | 1.39 (0.75-2.59) | 1.09 (0.42-2.79) |  | 0.73 (0.27-1.98) | 1.26 (0.45-3.51) |
| 71 - | Sales occupations | 1.73 (0.96-3.13) | **2.21 (1.19-4.12)** |  | 1.44 (0.79-2.61) | 1.76 (0.94-3.28) |  | 1.30 (0.72-2.36) | 1.55 (0.83-2.90) |  | 1.09 (0.55-2.15) | 1.71 (0.89-3.26) |
| 72 - | Customer service occupations | 1.42 (0.58-3.44) | 1.44 (0.51-4.04) |  | 1.30 (0.53-3.16) | 1.26 (0.45-3.55) |  | 1.18 (0.49-2.89) | 1.18 (0.42-3.32) |  | 1.08 (0.44-2.64) | 1.16 (0.41-3.26) |
| 81 - | Process, plant and machine operatives | **3.96 (1.90-8.24)** | 1.29 (0.72-2.30) |  | **2.96 (1.41-6.21)** | 1.08 (0.60-1.94) |  | **2.31 (1.10-4.87)** | 1.08 (0.60-1.93) |  | 2.08 (0.88-4.95) | 1.45 (0.77-2.74) |
| 811- | Process plant operatives | 1.60 (0.54-4.80) | **2.19 (1.11-4.30)** |  | 1.20 (0.40-3.60) | 1.79 (0.90-3.53) |  | 1.03 (0.34-3.11) | 1.83 (0.92-3.62) |  | 0.92 (0.28-3.07) | **2.39 (1.16-4.91)** |
| 82 - | Transport and mobile machine drivers and operatives | 1.22 (0.16-9.17) | 1.48 (0.88-2.47) |  | 1.01 (0.13-7.63) | 1.19 (0.71-2.01) |  | 0.88 (0.12-6.68) | 1.16 (0.69-1.94) |  | 0.57 (0.07-4.93) | 1.60 (0.86-3.00) |
|  |  |  |  |  |  |  |  |  |  |  |  |  |
| 8211 - | Large goods vehicle drivers | - | **2.34 (1.32-4.13)** |  | - | **2.03 (1.15-3.60)** |  | - | **2.07 (1.17-3.67)** |  | - | **2.69 (1.45-4.99)** |
| 8214 - | Taxi and cab drivers and chauffeurs | - | **3.48 (1.99-6.08)** |  | - | **2.83 (1.61-4.97)** |  | - | **2.48 (1.41-4.36)** |  | - | **3.46 (1.74-6.86)** |
| 9 - | Elementary occupations | **2.53 (1.20-5.35)** | 1.59 (0.97-2.60) |  | 2.03 (0.95-4.31) | 1.21 (0.73-2.00) |  | 1.82 (0.85-3.87) | 1.18 (0.72-1.96) |  | 1.51 (0.66-3.43) | 1.30 (0.77-2.20) |
| 91 - | Elementary trades and related occupations | **3.65 (1.61-8.26)** | 1.64 (0.93-2.90) |  | **2.59 (1.13-5.93)** | 1.15 (0.64-2.05) |  | 2.17 (0.95-4.97) | 1.10 (0.61-1.96) |  | 1.80 (0.63-5.11) | 1.70 (0.85-3.42) |
| 927 - | Other elementary services occupations | 1.18 (0.53-2.59) | **3.90 (2.03-7.52)** |  | 0.88 (0.40-1.95) | **2.59 (1.33-5.05)** |  | 0.76 (0.34-1.70) | **2.19 (1.12-4.26)** |  | 0.59 (0.23-1.49) | **2.44 (1.21-4.91)** |
| 9233 | Cleaners and domestics | **2.02 (1.07-3.80)** | 1.70 (0.61-4.79) |  | 1.43 (0.75-2.73) | 1.09 (0.38-3.10) |  | 1.20 (0.63-2.29) | 1.00 (0.35-2.83) |  | 0.95 (0.44-2.03) | 1.09 (0.38-3.13) |
| No code required | | **3.78 (1.96-7.27)** | **5.91 (3.61-9.67)** |  | **2.65 (1.39-5.05)** | **3.39 (2.01-5.70)** |  | 1.71 (0.89-3.26) | **3.14 (1.88-5.25)** |  | 2.02 (0.41-10.00) | **6.17 (2.47-15.41)** |

| *Housing tenure (reference category: Owned outright):* | | | | | | | | | | | |
| --- | --- | --- | --- | --- | --- | --- | --- | --- | --- | --- | --- |
| Owned with mortgage |  |  |  | 1.20 (0.82-1.75) | **1.61 (1.15-2.24)** |  | 1.12 (0.77-1.64) | **1.57 (1.12-2.19)** |  | 1.12 (0.76-1.63) | **1.55 (1.11-2.17)** |
| Social rented |  |  |  | **2.46 (1.66-3.65)** | **2.88 (2.02-4.10)** |  | **1.59 (1.06-2.36)** | **2.07 (1.45-2.95)** |  | **1.59 (1.07-2.37)** | **2.12 (1.48-3.03)** |
| Private rented |  |  |  | 1.49 (0.86-2.57) | **1.75 (1.10-2.78)** |  | 1.20 (0.70-2.08) | 1.47 (0.93-2.35) |  | 1.22 (0.71-2.11) | 1.50 (0.94-2.40) |
| Other/not known |  |  |  | **0.33 (0.14-0.77)** | 0.84 (0.42-1.69) |  | 1.33 (0.38-4.67) | **3.07 (1.63-5.77)** |  | 1.27 (0.36-4.46) | **3.06 (1.62-5.77)** |
| *Household size (UPRN; reference category: 1-2 people):* | | | | | | | | | | | |
| 3-4 people |  |  |  | 0.76 (0.57-1.01) | 0.95 (0.76-1.19) |  | 0.80 (0.60-1.07) | 1.00 (0.80-1.25) |  | 0.78 (0.58-1.04) | 0.96 (0.77-1.21) |
| 5-6 people |  |  |  | 0.70 (0.39-1.25) | 0.98 (0.63-1.53) |  | 0.73 (0.41-1.31) | 0.98 (0.63-1.53) |  | 0.68 (0.38-1.23) | 0.94 (0.60-1.46) |
| 7+ people |  |  |  | **3.67 (2.20-6.11)** | **5.27 (3.70-7.51)** |  | **3.48 (2.06-5.88)** | **4.20 (2.88-6.13)** |  | **2.84 (1.63-4.96)** | **3.78 (2.54-5.61)** |
| *Household with children (UPRN; reference category: No children):* | | | | | | | | | | | |
| At least one child (0-11 years) |  |  |  | 0.59 (0.32-1.09) | **0.35 (0.19-0.63)** |  | 0.58 (0.31-1.08) | **0.37 (0.20-0.66)** |  | 0.60 (0.33-1.12) | **0.37 (0.21-0.68)** |
| At least one child (12-17 years) |  |  |  | 0.79 (0.48-1.30) | **0.63 (0.41-0.97)** |  | 0.80 (0.49-1.31) | 0.67 (0.43-1.03) |  | 0.82 (0.50-1.35) | 0.68 (0.44-1.04) |
| *Whether multigenerational household (UPRN; reference category: No):* | | | | | | | | | | | |
| Yes |  |  |  | **2.06 (1.45-2.93)** | **1.83 (1.37-2.43)** |  | **1.94 (1.35-2.77)** | **1.73 (1.28-2.32)** |  | **1.80 (1.25-2.60)** | **1.65 (1.22-2.23)** |
| *Learning disability or difficulty (reference category: No):* | | | | | | | | | | | |
| Yes |  |  |  |  |  |  | **2.03 (1.21-3.41)** | **1.55 (1.03-2.33)** |  | **2.01 (1.20-3.36)** | **1.58 (1.05-2.37)** |
| Not known |  |  |  |  |  |  | **0.22 (0.05-0.95)** | **0.09 (0.04-0.21)** |  | **0.21 (0.05-0.92)** | **0.09 (0.04-0.20)** |
| *Whether shielding (reference category: No):* | | | | | | | | | | | |
| Yes |  |  |  |  |  |  | **4.93 (3.56-6.83)** | **4.91 (3.65-6.61)** |  | **4.95 (3.57-6.86)** | **4.95 (3.68-6.66)** |
| *Pre-pandemic health conditions (reference category for each covariate: No):* | | | | | | | | | | | |
| Cancer and immunosuppression |  |  |  |  |  |  | 1.67 (0.97-2.88) | 1.50 (0.94-2.39) |  | 1.66 (0.96-2.87) | 1.49 (0.94-2.38) |
| Cardiovascular conditions |  |  |  |  |  |  | **1.90 (1.42-2.54)** | **1.64 (1.31-2.05)** |  | **1.89 (1.42-2.53)** | **1.64 (1.31-2.05)** |
| Diabetes |  |  |  |  |  |  | **2.99 (2.26-3.96)** | **2.13 (1.70-2.67)** |  | **2.98 (2.25-3.95)** | **2.13 (1.70-2.67)** |
| Hypertension |  |  |  |  |  |  | 1.20 (0.91-1.56) | 1.19 (0.96-1.47) |  | 1.19 (0.91-1.56) | 1.19 (0.96-1.47) |
| Respiratory conditions |  |  |  |  |  |  | 1.13 (0.85-1.51) | 1.04 (0.81-1.34) |  | 1.14 (0.85-1.52) | 1.04 (0.81-1.34) |
| Other conditions |  |  |  |  |  |  | **1.46 (1.14-1.85)** | **1.80 (1.47-2.21)** |  | **1.45 (1.14-1.84)** | **1.80 (1.46-2.21)** |
| *Occupational exposure measures (O*NET scores 0-1):* | | | | | | | | | | | |
| Exposure to disease |  |  |  |  |  |  |  |  |  | **2.38 (1.21-4.71)** | **1.51 (1.01-2.26)** |
| Proximity to others |  |  |  |  |  |  |  |  |  | 2.40 (0.44-13.10) | 0.80 (0.30-2.16) |
| Environmentally controlled indoor conditions |  |  |  |  |  |  |  |  |  | 0.66 (0.13-3.42) | **2.47 (1.16-5.29)** |
|  |  |  |  |  |  |  |  |  |  |  |  |
| COVID-19 hospital admissions (n) | 300 | 460 |  | 300 | 460 |  | 300 | 460 |  | 300 | 460 |
| Total persons (n) | 859,800 | 840,870 |  | 859,800 | 840,870 |  | 859,800 | 840,870 |  | 859,800 | 840,870 |
| AIC | 7,968 | 12,189 |  | 7,887 | 12,034 |  | 7,659 | 11,748 |  | 7,658 | 11,744 |
| BIC | 8,260 | 12,527 |  | 8,295 | 12,488 |  | 8,173 | 12,306 |  | 8,206 | 12,338 |
| Likelihood Ratio (LR) test p-value | <0.001 | <0.001 |  | <0.001 | <0.001 |  | <0.001 | <0.001 |  | 0.022 | 0.050 |

^a^ Deaths occurring between 1 March 2020 and 31 January 2021.

^b^ Hazard Ratios (HR) and 95% Confidence Intervals (CI) are reported; Estimates are not reported if there were less than 10 deaths; HRs greater than 1 with p < 0.05 are highlighted in bold, while HRs less than 1 with p < 0.05 are shown in white within shaded grey cells.

**Table S8** The interactions between occupation and sex in the prediction of COVID-19 hospital admission and COVID-19 death for women relative to men aged 40-64 years in Scotland ^a^

|  | | COVID-19 hospital admission | |  | COVID-19 death | |
| --- | --- | --- | --- | --- | --- | --- |
|  | | Women vs. Men | |  | Women vs. Men | |
| Covariate | | HR (95%CI) ^b^ | |  | HR (95%CI) ^b^ | |
|  | |  | |  |  | |
| *Occupation (SOC 2010 codes)* | |  | |  |  | |
| 1 - | Managers, directors and senior officials | **0.73 (0.60-0.89)** |  |  | 0.72 (0.40-1.30) | |
| 2 - | Professional occupations | 0.82 (0.61-1.09) | |  | 0.73 (0.30-1.73) | |
| 22 - | Health professionals | 0.95 (0.72-1.25) | |  | 2.54 (0.33-19.66) | |
| 24 - | Business, media and public service professionals | **0.58 (0.39-0.86)** |  |  | - | |
| 3 - | Associate professional and technical occupations | **0.63 (0.47-0.84)** |  |  | **0.23 (0.08-0.70)** |  |
|  |  |  | |  |  | |
| 33 - | Protective service occupations | - | |  | - | |
| 35 - | Business and public service associate professionals | **0.70 (0.51-0.96)** |  |  | **0.24 (0.07-0.84)** |  |
|  |  |  | |  |  | |
| 41 - | Administrative occupations | 0.82 (0.65-1.03) | |  | **0.53 (0.30-0.94)** |  |
| 42 - | Secretarial and related occupations | - | |  | - | |
| 5 - | Skilled trades occupations | 0.68 (0.41-1.11) | |  | 0.54 (0.13-2.26) | |
| 53 - | Skilled construction and building trades | - | |  | - | |
| 543 - | Food preparation and hospitality trades | 0.95 (0.65-1.38) | |  | 0.45 (0.14-1.41) | |
| 6 - | Caring, leisure and other service occupations | 0.78 (0.57-1.08) | |  | 0.40 (0.15-1.12) | |
| 614 - | Caring personal services | **0.66 (0.47-0.92)** |  |  | 1.07 (0.23-4.87) | |
| 6145 - | Care workers and home workers | 0.79 (0.59-1.06) | |  | 0.95 (0.37-2.46) | |
| 71 - | Sales occupations | 0.83 (0.64-1.07) | |  | 0.63 (0.34-1.18) | |
| 72 - | Customer service occupations | 1.15 (0.78-1.72) | |  | 0.79 (0.23-2.71) | |
| 81 - | Process, plant and machine operatives | 1.23 (0.89-1.69) | |  | 1.51 (0.73-3.14) | |
| 811- | Process plant operatives | 0.93 (0.60-1.44) | |  | 0.42 (0.13-1.31) | |
| 82 - | Transport and mobile machine drivers and operatives | 0.74 (0.39-1.40) | |  | 0.56 (0.08-4.11) | |
|  |  |  | |  |  | |
| 8211 - | Large goods vehicle drivers | - | |  | - | |
| 8214 - | Taxi and cab drivers and chauffeurs | - | |  | - | |
| 9 - | Elementary occupations | **0.72 (0.52-0.98)** |  |  | 1.17 (0.60-2.30) | |
| 91 - | Elementary trades and related occupations | 1.03 (0.69-1.53) | |  | 1.37 (0.61-3.08) | |
| 927 - | Other elementary services occupations | 0.75 (0.54-1.05) | |  | **0.27 (0.12-0.64)** |  |
| 9233 | Cleaners and domestics | 1.41 (0.88-2.26) | |  | 0.89 (0.31-2.58) | |
| No code required | | 0.93 (0.80-1.08) | |  | **0.46 (0.31-0.67)** |  |
|  | |  | |  |  | |
| Likelihood Ratio (LR) test p-value | | 0.015 | |  | 0.014 | |

^a^ Hospital admissions and deaths occurring between 1 March 2020 and 31 January 2021.

^b^ Hazard Ratios (HR) and 95% Confidence Intervals (CI) from Cox Proportional Hazards models including interactions between occupation and sex; Other covariates (not shown here) were those included in Model 4 for COVID-19 hospital admission and death, reported in Tables S6 and S7, respectively; Estimates are not reported if there were less than 10 hospital admissions or deaths; HRs greater than 1 with p < 0.05 are highlighted in bold, while HRs less than 1 with p < 0.05 are shown in white within shaded grey cells.
